# Extended infusion of β-lactams significantly reduces mortality and enhances microbiological eradication in paediatric patients: a systematic review and meta-analysis

**DOI:** 10.1016/j.eclinm.2023.102293

**Published:** 2023-11-02

**Authors:** Kinga Anna Budai, Ágnes Eszter Tímár, Mahmoud Obeidat, Vanda Máté, Rita Nagy, Andrea Harnos, Szilvia Kiss-Dala, Péter Hegyi, Miklós Garami, Balázs Hankó, Csaba Lódi

**Affiliations:** a University Pharmacy, Department of Pharmacy Administration, Semmelweis University, Budapest, Hungary; b Centre for Translational Medicine, Semmelweis University, Budapest, Hungary; c Heim Pál National Pediatric Institute, Budapest, Hungary; d Pediatric Center, MTA Center of Excellence, Semmelweis University, Budapest, Hungary; e Institute for Translational Medicine, Medical School, University of Pécs, Pécs, Hungary; f Department of Biostatistics at the University of Veterinary Medicine, Budapest, Hungary; g Institute of Pancreatic Diseases, Semmelweis University, Budapest, Hungary

**Keywords:** Beta-lactam, Extended infusion, Continuous infusion, Paediatric patients, Mortality

## Abstract

**Background:**

Paediatric patients are often exposed to subtherapeutic levels or treatment failure of β-lactams, and prolonged infusion may be beneficial. We aimed to investigate the efficacy and safety of extended infusion (EI; defined as ≥3 h) or continuous infusion vs. short, intermittent infusion (SI; defined as ≤60 min) of β-lactams in patients <21 years of age.

**Methods:**

A systematic review and meta-analysis was conducted to compare EI and continuous infusion with SI of β-lactams in children. A systematic search was performed in MEDLINE (via PubMed), Embase, CENTRAL, and Scopus databases for randomised controlled trials (RCTs) and observational studies published from database inception up to August 22, 2023. Any comparative study concerned with mortality, clinical efficacy, adverse events, or plasma concentrations of β-lactams for any infection was eligible. Case reports, case series, and patients aged >21 years were excluded. Odds ratios (OR) and median differences with 95% confidence intervals (CI) were calculated using a random-effects model. Risk of bias (ROB) was assessed using ROB2 and ROBINS-I tools. The protocol was registered with PROSPERO, CRD42022375397.

**Findings:**

In total, 19,980 articles were screened, out of which 19 studies (4195 patients) were included in the meta-analysis. EI administration was associated with a significantly lower all-cause mortality in both RCTs and non-RCTs [OR 0.74; CI 0.55–0.99; I^2^ = 0%; CI 0–58%]. Early microbiological eradication was higher with EI [OR 3.18; CI 2.24–4.51; I^2^ = 0%; CI 0–90%], but the clinical cure did not differ significantly between the two groups [OR 1.20; CI 0.17–8.71; I^2^ = 79%; CI 32–93%]. Achieving the optimal plasma level (50–100% fT > MIC) appeared favourable in the EI group compared to the SI. No significant differences were observed in the adverse events. The overall ROB was high because of the small sample sizes and clinically heterogeneous populations.

**Interpretation:**

Our findings suggest that extended infusion of β-lactams was associated with lower mortality and increased microbiological eradication and was considered safe compared to short-term infusion.

**Funding:**

None.

**Research in context**

**Evidence before this study**

Meta-analyses among adults and pharmacokinetic studies and reviews among paediatric patients support the hypothesis that extended or continuous infusion of beta-lactams can be more efficacious than the standard intermittent infusion. A literature search of four databases (MEDLINE via PubMed, Embase, CENTRAL, and Scopus) was performed on the 22nd of August 2023. No language or other restrictions were applied. The search key contained one paediatric, two dosing, and one antibiotic domain. Patients under the age of 21 years were included, and comparative studies were eligible that contained information regarding mortality, clinical efficacy, adverse events, or plasma concentrations of β-lactams. The overall risk of bias was high because of the small number of studies and complex composition of the study population, characterised by diverse age groups and comorbid conditions.

**Added value of this study**

This is the first comprehensive meta-analysis investigating the clinical efficacy and safety of extended infusion of β-lactams in paediatric patients. Our study provides data that extended infusion of beta-lactams showed significantly lower all-cause mortality and earlier microbiological eradication than short-term infusion, and it was associated with a lower infection-related mortality rate in children. Furthermore, there was no significant difference in adverse events. The study not only reported statistical significance but also considered clinical relevance, which is crucial for translating research findings into clinical practice. Our findings encourage further prospective data collection of extended infusion especially among neonates and critically ill children due to the limitations and low evidence level.

**Implications of all the available evidence**

Considering extended or continuous infusion of beta-lactams, particularly for neonates and critically ill children, as well as for patients receiving meropenem, is advisable. The implementation of therapeutic drug monitoring is essential. To gain a more precise understanding of the issue at hand, further prospective data collection through comparative clinical trials, along with the measurement of plasma concentrations, is warranted.

## Introduction

Parenteral beta-lactams, including penicillins, cephalosporins, carbapenems, and monobactams, are commonly used antibiotics in paediatrics. Various modes of administration are employed: 1) standard, intermittent (SI) or bolus infusion over 30–60 min, 2) extended infusion (EI) over 3–4 h, 3) continuous infusion over 24 h. In order to provide the best care, we must understand which regimen is better.

Furthermore, the misuse of antibiotics is a serious problem in medical practice, it can lead to treatment failure and increasing the risk of developing antibiotic resistance.^1^ To prevent antibiotic resistance, it is essential to maintain plasma concentrations in the target range. Beta-lactams are time-dependent antibiotics, and the optimal microbiological response relies on the duration during which the level of unbound drug remains above the minimal inhibitory concentration (MIC): fT > MIC.^2^ In critically ill patients, it is recommended to maintain plasma concentrations 1- or 4-fold higher than the MIC throughout the dosing interval (100% fT > 1–4 × MIC).^3^

Inadequacy of the standard administration was confirmed by a multicentre pharmacokinetic point-prevalence study: only 127 of 361 (35.2%) critically ill adult patients achieved 100% fT > 4 × MIC target receiving one beta-lactam antibiotic.^4^ Subtherapeutic concentrations were measured in 117 of 157 (74.5%) patients treated with SI of amoxicillin-clavulanic acid, piperacillin-tazobactam, and meropenem in a paediatric intensive care unit (PICU). Using the pharmacokinetic/pharmacodynamic (PK/PD) target of 100% fT > 4 × MIC, only 12 of 157 (7.6%) patients achieved the required level.^5^ A decreased probability of target attainment was observed in several PK studies in critically ill populations for various antibiotics with standard dosages, and the benefits of prolonged infusion were described.^6^^,^^7^

EI is strongly suggested in adults due to the probable clinical advantages. A meta-analysis of randomised controlled trials (RCTs) demonstrated that EI of antipseudomonal beta-lactams for treating sepsis was resulted in significantly lower mortality than SI.^8^ Furthermore, all-cause mortality was lower in critically ill adult patients with predominant respiratory infections who received EI than in those who received SI.^9^

Based on this, we may assume that paediatric patients, especially critically ill patients, are similarly affected by inappropriate dosing and that EI might be similarly beneficial for them. Our aim, therefore, was to investigate the clinical efficacy and safety of EI for beta-lactams in children.

## Methods

### Search strategy and selection criteria

The recommendations of the Preferred Reporting Items for Systematic Reviews and Meta-Analyses (PRISMA) 2020 guidelines,^10^ and the Cochrane Handbook^11^ were followed. The study protocol was registered in the PROSPERO database (CRD42022375397). We conducted minor changes: infection-related mortality was examined because it can represent the real effectiveness of the administered drug; and patients were under the age of 18 instead of 21 years in the included articles, because studies with a population between age of 18 and 21 years were not identified.

The PICO framework^12^ was used to answer the clinical question. Studies reporting on (P) paediatric patients (0–21 years) treated with beta-lactam antibiotics were included. In the intervention group (I), prolonged, extended, or continuous infusion was administered; the comparator group (C) received short-term and intermittent infusions. In the following, we refer to EI and continuous infusion collectively as extended. The primary outcomes (O) were all-cause, short-term intra-hospital mortality, and infection-related mortality. As secondary outcomes, we evaluated the rate of clinical cure or treatment failure, success of microbiological eradication, appearance of adverse events, achievement of PK/PD targets based on drug levels, and length of hospital stay (LOS). RCTs, non-randomized observational studies (non-RCTs) were included in the analysis. Adult patients (>21 years of age), case reports, and case series were excluded.

Our systematic search was conducted on the 27th of November 2022, and an updated search on the 22nd of August 2023, using four medical databases (MEDLINE via PubMed, Embase, CENTRAL, and Scopus). In addition, the references of the included studies were searched.

During the systematic search, the search key contained four domains without language restrictions or filtering options: one paediatric, two dosing, and one antibiotic domain. The full search key is provided in Supplementary Material (Supplementary Table 1S).

The search results were exported to EndNote 20 citation manager (Clarivate Analytics, Philadelphia, PA, USA). After automatic and manual duplicate removal (KAB), articles were selected using the Rayyan Intelligent Systematic Review program. The selection was performed by two independent review authors (KAB and ÁET) by title, abstract, and full text according to the inclusion criteria. Disagreements were resolved with the corresponding author’s involvement. Cohen’s Kappa coefficient was calculated at each selection step to evaluate the level of agreement.

### Data analysis

From the eligible articles, patient-level data were independently collected by two authors (KAB and ÁET) using a standardized data collection sheet.

The following data were extracted: study characteristics (first author, year of publication, study design, study period, country, number of centres, name of drugs), population description and demographics (sample size, percentage of female participants, age, indication of the antibiotic(s), patients with positive microbiological culture, patients with co-administered antibiotics), therapy details (drug type, dose, regimen, length of infusion, duration), and outcomes as reported in each article. Microsoft Excel (Microsoft, Office 365, Redmond, WA, USA) was used for data collection.

Two authors (KAB, ÁET) independently performed the risk of bias assessment using the RoB2 risk-of-bias tool in the case of RCTs and the ROBINS-I tool in the case of non-RCTs. A consensus was reached by involving the corresponding author. The domains evaluated were bias arising from the randomisation process (RCTs), bias due to confounding (non-RCTs), selection of participants (non-RCTs), classification of intervention (non-RCTs), deviations from the intended intervention (both), missing data (both), the measurement of the outcomes (both), and the selection of the reported results (both). The conclusion of the risk assessment was characterized as ‘low’, ‘moderate’ and ‘serious’.

Grading of Recommendations Assessment, Development and Evaluation (GRADE)^13^ approach was followed to evaluate the quality of evidence of our results, and the GRADEpro tool (software; McMaster University and Evidence Prime, 2022. Available from gradepro.org) was used based on the recommendations of the Cochrane Collaboration. Each outcome was rated for the risk of bias, inconsistency, indirectness, imprecision, publication bias, and the presence of a large effect, dose-dependent response. Plausible confounders were rated as ‘not serious’, ‘serious’, or ‘very serious’. The final certainty of evidence was categorised as ‘very low’, ‘low’, ‘moderate’, or ‘high’.

Considering the study heterogeneity, a random-effects model was used to pool effect sizes. Odds ratios (OR) and risk ratios (RR) with 95% confidence intervals (CIs) were calculated for dichotomous outcomes extracted based on the numbers of patients and events extracted from the studies. The results are presented as the odds or risk of an event in the extended group divided by the odds or risk of the same event in the bolus group. Pooled risk ratios were calculated for all-cause mortality if all meta-analysed studies were RCTs.

For continuous outcomes (LOS, PICU length of stay, and duration of antibiotic course), quartiles were given in most cases (instead of mean and standard deviation). The difference between the group medians was used as an effect size measure with 95% CI.^14^ Median values of the bolus group were subtracted from those of the extended group.

The results were considered statistically significant if the pooled CI did not contain the null value, and p-value was less than 0.05. We summarised the findings related to the meta-analysis on forest plots. If the study number was sufficiently large and not too heterogeneous, we reported the prediction intervals (i.e., the expected range of effects of future studies) of the results where applicable. Between-study heterogeneity was described using Higgins and Thompson’s I^2^ statistics.^15^

Small study publication bias was assessed by visual inspection of funnel plots. Potential outlier publications were explored by using different influence measures and plots.^16^

All statistical analyses were calculated by *R software*^17^ using the *meta*^18^ package for OR meta-analysis calculations and plots, the *metamedian*^19^ package for difference of median meta-analysis calculations, and the *dmetar*^20^ package for additional influential analysis calculations and plots.

A subgroup analysis was performed on all-cause mortality (RCTs and non-RCTs), infection-related mortality (non-RCTs), and acute kidney injury (neonates and patients with cystic fibrosis [CF]). We analysed the all-cause mortality of neonates and meropenem-treated patients separately.

Additional details on calculations, data synthesis, publication bias assessment, and influential analyses, are included in the Supplementary Material (pages 3–4).

### Role of the funding source

There was no funding source for this study. The corresponding author and KAB had access to all the data and had responsibility for the decision to submit the study for publication.

## Results

### Search and selection

**Fig. 1:**
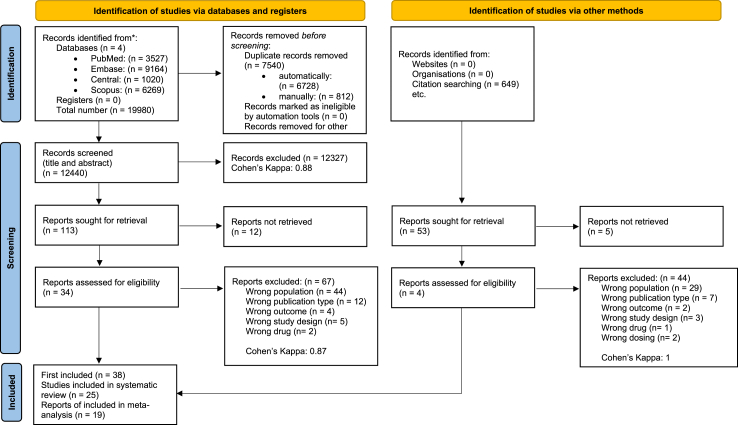
PRISMA flowchart of the article selection process.

In total, 19,980 studies were identified and screened. After duplicate removal, and title and abstract selection (Cohen’s Kappa 0.88), we found 34 eligible articles during the full-text article analysis (Cohen’s Kappa 0.87). Three other eligible articles were identified through citation checking. One additional Chinese study^21^ was selected from a systematic review^7^ and extracted with XL’s help. Nineteen of 38 articles were excluded from the meta-analysis because of overlapping populations (6), no comparator (6), or their outcomes could not be pooled with others (7). Overall, 25 studies were included in our systematic review and 19 in our meta-analysis.^3^^,^^21^, ^22^, ^23^, ^24^, ^25^, ^26^, ^27^, ^28^, ^29^, ^30^, ^31^, ^32^, ^33^, ^34^, ^35^, ^36^, ^37^, ^38^ One of them was a trial protocol^26^ and one of them was poster abstract.^30^ Two studies were bicentric,^26^^,^^35^ the others were single centre. The studies included patients under 18 years of age. The selection process is summarised in Fig. 1. The excluded studies at full-text screening stage are listed in Supplementary Table 2S.

### Basic characteristics of included studies

The included 19 studies consisted of 4195 patients from USA (7),^23^^,^^27^^,^^30^, ^31^, ^32^^,^^36^^,^^38^ China (2),^21^^,^^34^ Thailand (2),^22^^,^^24^ France (1),^3^ Mexico (2),^25^^,^^26^ Angola (2),^28^^,^^29^ Estonia (1),^35^ Switzerland (1),^37^ Egypt (1).^33^ The underlying diseases were suspected or proven Gram-negative bacteraemia (GNB), sepsis, neonatal sepsis, meningitis, exacerbation of CF, and febrile neutropenia. The administered beta-lactams were cefazolin, cefepime, cefotaxime, ceftazidime, imipenem, meropenem, and piperacillin/tazobactam. The baseline characteristics are summarised in Table 1, Table 2, Table 3. The dosing is detailed in Table 4. The hypotheses of the RCTs for calculating the number of patients are in the Supplementary Material (page 9).

**Table 1:** Baseline characteristics of the included trials in the meta-analysis except neonatal studies.

Study	Country	Study design	Population	Renal function (CrCl) median [IQR] mL/min/1.73 m^2^	Extracorporeal therapy (CKRT, ECMO)	Sample size (% female)	Antibiotic(s)	Inter-vention group [EI]	Sample size [EI group] (% female)	Median age [IQR] (years) [EI group]	Control group [SI]	Sample size [SI group] (% female)	Median age [IQR] (years) [SI group]	Outcomes
Beauchamp et al. (2019)^36^	USA	Retro-spective cohort	Proven GNB	NA	NA	67 (38.8)	Cefepime	EI, 4 h	21 (42.9)	7 [0.82; 11.5]	0.5 h	46 (37)	0.5 [0.2; 2.2]	Treatment failure; all-cause mortality; infection-related mortality; LOS; PICU LOS; duration of the antibiotic; all adverse events
Chongcharoenyanon et al. (2021)^22^	Thailand	RCT	Suspected or proven MDR-GNB infection	NA	Excluded	90 (56.7)	Piperacillin/tazobactam	EI, 4 h	45 (60.0)	4.83 [1.75; 10.8]	0.5 h	45 (53.3)	2.67 [1.08; 7.5]	Optimal exposure (50% fT > MIC and 50% fT > 4 × MIC); all-cause mortality; treatment failure; LOS
Chosidow et al. (2020)^3^	France	Prospective observational	PICU	CrCl: 167 [117; 190]	CKRT: 3, ECMO: 3	37 (64.9)	Cefotaxime, piperacillin/tazobactam, amoxicillin, meropenem, cefazolin, imipenem, ceftazidime	CI	4 (NA)	NA	NA (SI)	33 (NA)	NA	Optimal exposure (100% fT > 4 × MIC); all adverse events
Imburgia et al. (2022)^23^	USA	Retro-spective cohort	CF	NA	NA	188 (51.6)	Cefepime	EI, 4 h	135 (52)	12 [6; 16]	0.5 h	53 (51)	12 [6; 16]	AKI; hepatic adverse events; all adverse events; LOS
Maimongkol et al. (2022)^24^	Thailand	Prospective observational	Suspected or proven MDR-GNB infection	CrCl: 123.1 [94.1; 159.2]	Excluded	72 (55.6)	Meropenem	EI, 3 h	54 (53.7)	1 [0.333; 3]	0.5–1 h	18 (61.1)	0.917 [0.25; 3.33]	Optimal exposure (50% fT > MIC; 100% fT > MIC); all-cause mortality; microbiological eradication; LOS; all adverse events
NCT03019965 clinical trial (Fuentes, 2016)^26^	Mexico	RCT	Sepsis	Patients with chronic kidney disease or acute renal failure were excluded	NA	403 (45.2)	Imipenem, meropenem, piperacillin/tazobactam	CI	202 (45)	6 [NA]	0.5–1 h	201 (45.3)	6 [NA]	Clinical cure; all-cause mortality; all adverse events
Nichols et al. (2015)^27^	USA	Prospective observational	Infection	The dosing was adjusted for impaired renal function	NA	150 (48)	Cefepime	EI, 4 h	143 (NA)	NA	0.5	7 (NA)	NA	Duration of antibiotic; LOS
Pelkonen et al. (2011)^29^	Angola	RCT	Bacterial meningitis	NA	NA	723 (46.6)	Cefepime	CI for 24 h, then SI	183 (41.5)	1.42 [NA]	NA (SI)	180 (50)	0.917 [NA]	Treatment failure; all-cause mortality; all adverse events
Rappaz et al. (2000)^37^	Switzerland	Prospective observational	CF	NA	NA	14 (57.1)	Ceftazidime	CI	14 (57.1)	14.8 [10.1; 15.3]	0.333 h	14 (57.1)	14.8 [10.1; 15.3]	All adverse events
Repholz et al. (2017)^30^	USA	Retro-spective observational	CF	NA	NA	113 (NA)	Meropenem, aztreonam, piperacillin/tazobactam, cefepime	NA (EI)	100 (NA)	NA	NA (SI)	13 (NA)	NA	AKI; hepatic adverse events; LOS
Riggsbee et al. (2023)^38^	USA	Retro-spective cohort	CF	Normal baseline creatinine levels	NA	204 (NA)	Piperacillin/tazobactam	EI, 4 h	95 (NA)	7 [3; 12]	0.5 h	109 (NA)	8 [4; 13]	AKI; hepatic adverse events; clinical cure; duration of antibiotic; LOS
Savonius et al. (2021)^28^	Angola	RCT	Bacterial meningitis	NA	NA	373 (42.1)	Cefotaxime	CI for 96 h, then SI	187 (46)	2.33 [NA]	NA (SI)	186 [38.2]	2.58 [NA]	All-cause mortality; LOS
Solórzano-Santoz et al. (2019)^25^	Mexico	RCT	Cancer, FN	Patients with chemotherapy-associated nephrotoxicity were excluded	NA	176 (41.5)	Piperacillin/tazobactam	CI	76 (38.2)	10 [NA]	0.5 h	100 (44)	9 [NA]	Treatment failure; all-cause mortality
Zembles et al. (2021)^32^	USA	Retro-spective chart analysis	Oncology, bone narrow transplant, critical care, general surgery	The dosing was adjusted for impaired renal function	CKRT: excluded, ECMO: 25	551 (40.7)	Cefepime, meropenem, piperacillin/tazobactam	EI, 3–4 h	258 (43)	7.2 [2.8; 13.7]	0.25–0.5 h	293 (38.6)	8.3 [3.1; 13]	All-cause mortality; LOS; duration of antibiotic
Zembles et al. (2022)^31^	USA	Retro-spective chart analysis	Proven GNB	NA	NA	124 (29.8)	Cefepime, meropenem, piperacillin/tazobactam	EI, 3–4 h	51 (27.5)	1.7 [1; 10.5]	≤0.5 h	73 (31.5)	5 [2.1; 11.5]	All-cause mortality; LOS; duration of antibiotic

AKI, acute kidney injury; CKRT, continuous kidney replacement therapy; ECMO, extracorporeal membrane oxygenation; CF, cystic fibrosis; CI, continuous infusion; CrCl, creatinine clearance; EI, extended infusion; FN, febrile neutropenia; GNB, Gram-negative bacteremia; h, hours; IQR, interquartile range; LOS, length of hospital stay; MDR, multidrug-resistant; MIC, minimal inhibitory concentration; NA, not reported or not applicable; PICU, paediatric intensive care unit; RCT, randomised controlled trial, SI, short-term intermittent infusion.

**Table 2:** Baseline characteristics of the included neonatal studies in the meta-analysis.

Study	Country	Study design	Population	Renal function	Extracorporeal therapy (CKRT, ECMO)	Sample size (% female)	Antibiotic(s)	Inter-vention group [EI]	Sample size [EI group] (% female)	Mean PNA at sampling ± SD (days) [EI group]	Control group [SI]	Sample size [SI group] (% female)	Mean PNA at sampling ± SD (days) [SI group]	Outcomes
Cao et al. (2022)^34^	China	Retro-spective cohort	Neonatal sepsis	NA	NA	256 (NA)	Meropenem	EI, 2–3 h	127 (NA)	NA	0.5 h	129 (NA)	NA	Treatment failure; microbiological eradication; AKI; hepatic adverse events
Padari et al. (2012)^35^	Estonia	Prospective observational	Neonatal sepsis	Normal baseline creatinine levels	NA	19 (36.8)	Meropenem	EI, 4 h	10 (40)	20.5 ± 6.6	0.5 h	9 (33.3)	15.6 ± 8.6	All-cause mortality; optimal exposure; (100% fT > MIC); duration of antibiotic; all adverse events
Shabaan et al. (2017)^33^	Egypt	RCT	Neonatal sepsis	Patients with renal failure were excluded	NA	102 (46.1)	Meropenem	EI, 4 h	51 (51)	8 [6; 13]‡	0.5 h	51 (41.2)	6 [5; 15]‡	All-cause mortality; all adverse events; clinical cure; microbiological eradication; AKI; hepatic adverse events; LOS
Wang et al. (2018)^21^	China	RCT	Neonatal sepsis	Normal baseline creatinine levels	NA	120 (44.2)	Meropenem	EI, 3 h	60 (50)	11.7 ± 2.3	0.5 h	60 (38.3)	13.2 ± 3.2	All-cause mortality; AKI

AKI, acute kidney injury; CKRT, continuous kidney replacement therapy; ECMO, extracorporeal membrane oxygenation; EI, extended infusion; h, hours; IQR, interquartile range; LOS, length of hospital stay; MIC, minimal inhibitory concentration; NA, not reported or not applicable; PNA, postnatal age; RCT, randomised controlled trial; SD, standard deviation; SI, short-term intermittent infusion.

a Data expressed as median [interquartile range].

**Table 3:** Baseline characteristics of the included studies in the systematic review.

Study	Country	Study design	Population	Renal function (CrCl) median [IQR] mL/min/1.73 m^2^	Extracorporeal therapy (CKRT, ECMO)	Sample size (% female)	Antibiotic(s)	Inter-vention group [EI]	Sample size [EI group] (% female)	Median age [IQR] (years) [EI group]	Control group [SI]	Sample size [SI group] (% female)	Median age [IQR] (years) [SI group]
André et al. (2021)^39^	Switzerland	Retrospective cohort	Cancer, FN	NA^a^	NA	32 (NA)	Meropenem, piperacillin	CI	3 (NA)	NA	0.5 h	32 (NA)	NA
Debray et al. (2022)^40^	France	Prospective observational	PICU	143 [85; 184],^b^	CKRT: 21^b^ ECMO: NA	80 (38.7), 106 antibiotic courses	Cefotaxime, piperacillin/tazobactam, meropenem	CI	32 (34.4)^b^	2 [0.567; 12]^b^	0.5 h	74 (40.5)^b^	1.58 [0.608; 9]^b^
Gatti et al. (2023)^41^	Italy	Retrospective cohort	PICU	139.6 [91.8; 171.5]	CKRT: 1 ECMO: NA	21 (52.3)	Ceftazidime, meropenem, piperacillin/tazobactam	CI	21 (52.3)	2 [1; 8]	NA	NA	NA
Knoderer et al. (2017)^42^	USA	Retrospective cohort	Gram-negative infection	NA	CKRT: excluded ECMO: NA	39 (51.3)	Piperacillin/tazobactam	EI, 4 h	39 (51.3)	NA	NA	NA	NA
Ragonnet et al. (2023)^43^	France	Retrospective cohort	PICU	NA	NA	175 (NA)	NA	CI	72 (NA)	NA	SI	103 (NA)	NA
van Boekholt et al. (2016)^44^	The Netherlands	Pharmaco-kinetic study	Neonatal infection	NA^c^	NA	11 (63.6)	Amoxicillin	CI	11 (63.6)	NA^d^	NA	NA	NA

CKRT, continuous kidney replacement therapy; CrCl, creatinine clearance; CI, continuous infusion; ECMO, extracorporeal membrane oxygenation; EI, extended infusion; FN, febrile neutropenia; h, hours; IQR, interquartile range; NA, not reported or not applicable; PICU, paediatric intensive care unit; SD, standard deviation; SI, short-term intermittent infusion.

a Patients with augmented renal clearance (ARC, CrCl >160 mL/min/1.73 m^2^), with normal renal function (CrCl 80–160 mL/min/1.73 m^2^) and with renal insufficiency (CrCl <80 mL/min/1.73 m^2^) were included.

b Based on antibiotic courses.

c The serum creatinine (μmol/L) of the included patients was mean (SD) 74.4 (23.6), but they were not able to calculate neonatal GFR because of the maternal origin of the creatinine.

d Gestational age, days; mean (SD) 265.5 (22.0).

**Table 4:** Summary of the applied beta-lactam therapies in the studies included in the meta-analysis.

Study	Antibiotic	Coadministered antibiotic (°N of patients)	Dose Median [IQR] (range min–max) [intervention group]	Regimen [intervention group]	Coadministered antibiotic (°N of patients) [intervention group]	Dose Median [IQR] (range min–max) [control group]	Regimen [control group]	Coadministered antibiotic (°N of patients) [control group]
Chosidow et al. (2020)^3^	Cefazolin	9	150 (75–300) mg/kg/d	CI	NA	50 (25–100) mg/kg	q8 h	NA
Beauchamp et al. (2019)^36^	Cefepime	29	50 [31.5–50.3] mg/kg	20: q8 h 1: q24 h	3	50 [46.8–50.1] mg/kg	1: q6 h 23: q8 h 20: q12 h 2: q24 h	26
Imburgia et al. (2022)^23^	Cefepime	51	48.4 [40.6–50] mg/kg	q8 h	38	48.8 [40.4–50] mg/kg	q8 h	13
Nichols et al. (2015)^27^	Cefepime	80	50 mg/kg	q8 h	76	50 mg/kg	q8 h	4
Zembles et al. (2021)^32^	Cefepime	NA	50 mg/kg	q8 h	NA	50 mg/kg	q8 h	NA
Zembles et al. (2022)^31^	Cefepime	NA	50 mg/kg	q8 h	NA	50 mg/kg	q8 h	NA
Pelkonen et al. (2011)^29^	Cefotaxime	87	250 mg/kg/d (q12 h) for 24 h, then 62.5 mg/kg	CI for 24 h, then q6 h	46	62.5 mg/kg	q6 h	41
Savonius et al. (2021)^28^	Cefotaxime	NA	250 mg/kg/d (q12 h) for 96 h, then 62.5 mg/kg	CI for 96 h, then q6 h	NA	62.5 mg/kg	q6 h	NA
Rappaz et al. (2000)^37^	Ceftazidime	14	100 mg/kg/d	CI	14	200 mg/kg/d	q8 h	14
NCT03019965 clinical trial (Fuentes, 2016)^26^	Imipenem	NA	20 mg/kg bolus (1 h), then 80 mg/kg/d	CI	NA	20 mg/kg	q6 h	NA
Cao et al. (2022)^34^	Meropenem	NA	20 mg/kg	q8 h	NA	20 mg/kg	q8 h	NA
Padari et al. (2012)^35^	Meropenem	7	20 mg/kg bolus (0.5 h), then 40 mg/kg/d 18.3 ± 2.4^a^	q12 h	3	20 mg/kg 18.9 ± 5^a^	q12 h	4
Maimongkol et al. (2022)^24^	Meropenem	29	NA (38–40) mg/kg	q8 h	23	NA (20–21) mg/kg	q8 h	6
NCT03019965 clinical trial (Fuentes, 2016)^26^	Meropenem	NA	35 mg/kg bolus (1 h), then 100 mg/kg/d	CI	NA	33.3 mg/kg	q8 h	NA
Shabaan et al. (2017)^33^	Meropenem	NA	NA (20–40) mg/kg	q8 h	NA	NA (20–40) mg/kg	q8 h	NA
Wang et al. (2018)^21^	Meropenem	NA	20 mg/kg	q8 h	NA	20 mg/kg	q8 h	NA
Zembles et al. (2021)^32^	Meropenem	NA	NA (20–40) mg/kg	q8 h	NA	NA (20–40) mg/kg	q8 h	NA
Zembles et al. (2022)^31^	Meropenem	NA	NA (20–40) mg/kg	q8 h	NA	NA (20–40) mg/kg	q8 h	NA
Chongcharoenyanon et al. (2021)^22^	Piperacillin/tazobactam	0	100 mg/kg bolus (0.5 h), then 100 mg/kg	q8 h	0	100 mg/kg	q8 h	0
NCT03019965 clinical trial (Fuentes, 2016)^26^	Piperacillin/tazobactam	NA	75 mg/kg bolus (0.5 h), then 300 mg/kg/h	CI	NA	75 mg/kg	q6 h	NA
Riggsbee et al. (2023)^38^	Piperacillin/tazobactam	NA	443.7 [412.1–450] mg/kg/d	NA	NA	401.5 [392–449.8] mg/kg/d	NA	NA
Solórzano-Santoz et al. (2019)^25^	Piperacillin/tazobactam	0	75 mg/kg bolus (0.5 h), then 100 mg/kg	q8 h	0	100 mg/kg	q8 h	0
Zembles et al. (2021)^32^	Piperacillin/tazobactam	NA	NA (75–100) mg/kg	q8 h	NA	100 mg/kg	q8 h	NA
Zembles et al. (2022)^31^	Piperacillin/tazobactam	NA	NA (75–100) mg/kg	q8 h	NA	100 mg/kg	q8 h	NA

CI, continuous infusion; d, day; h, hour; IQR, interquartile range; kg, kilogram; mg, milligram; °N, number; NA, not reported or not applicable; q, every.

Repholz et al. (2017): no data about dose and regimen.

a Data expressed as mean ± standard deviation.

### Primary outcome

#### Decrease in mortality

**Fig. 2:**
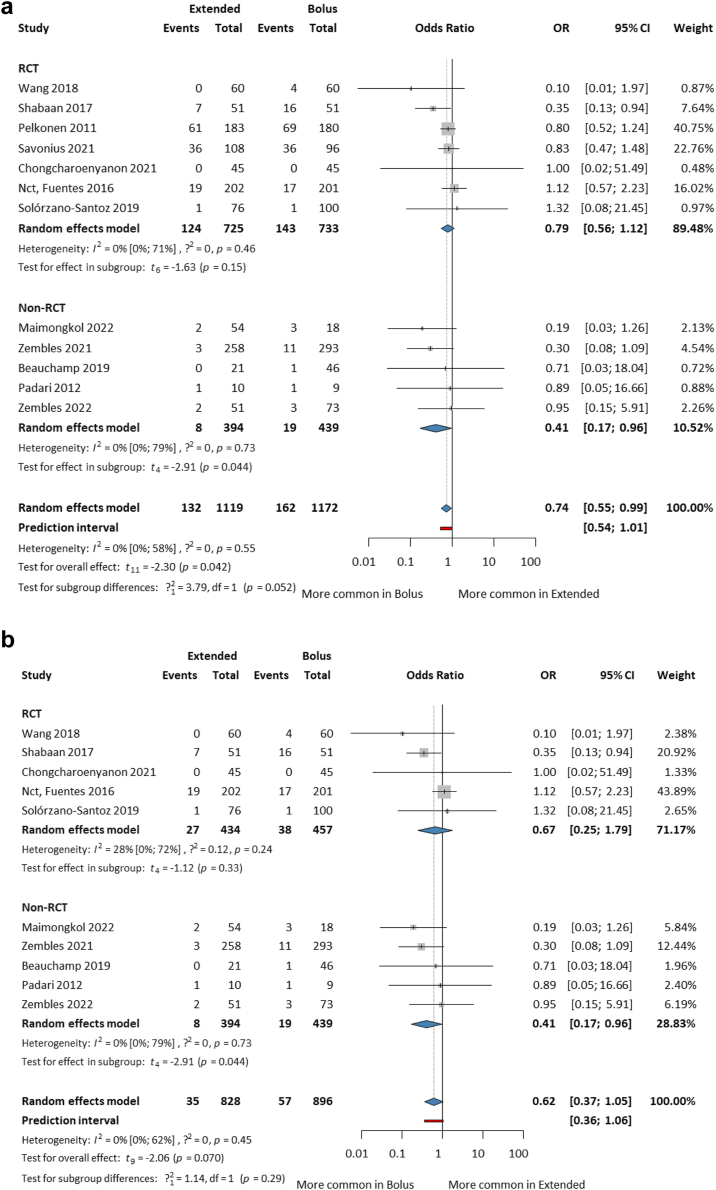
Forest plots of mortality among paediatric patients treated with extended vs. bolus infusion of beta-lactams. (a) All studies (b) without meningitis studies.

Seven RCTs and five non-RCTs that reported all-cause mortality within 30 days from the beginning of treatment or in-hospital mortality were included. Separate analyses are in the Supplementary Material (Supplementary Figures 1aS and bS and 2S). Overall, EI was associated with a significantly lower mortality than SI [11.8% vs. 13.8%; OR 0.74; CI 0.55–0.99; I^2^ = 0%; CI 0–58%]. Non-RCTs showed a significant effect, and RCTs had the similar, but non-significant tendency. With the two meningitis studies^28^^,^^29^ omitted because of the different routes of administration and extremely severe conditions, clinically relevant but statistically insignificant results were obtained [4.62% vs. 7.17%; OR 0.62; CI 0.37–1.05; I^2^ = 0%; CI 0–62%] (Fig. 2a and b). The population was mixed, but statistically not heterogeneous. Funnel plots, influential analyses and leave-one-out plots are in the Supplementary Material (Supplementary Figures 7S–9S). No sign for publication bias was observed. Two articles^26^^,^^29^ as potential influentials are detected, although their influential effect is mainly due to the large number of participants. The change in effect size would be not clinically meaningful by omitting Pelkonen et al.,^29^ but it would be by omitting Fuentes et al.^26^ (after omitting the two meningitis studies^28^^,^^29^).

**Fig. 3:**
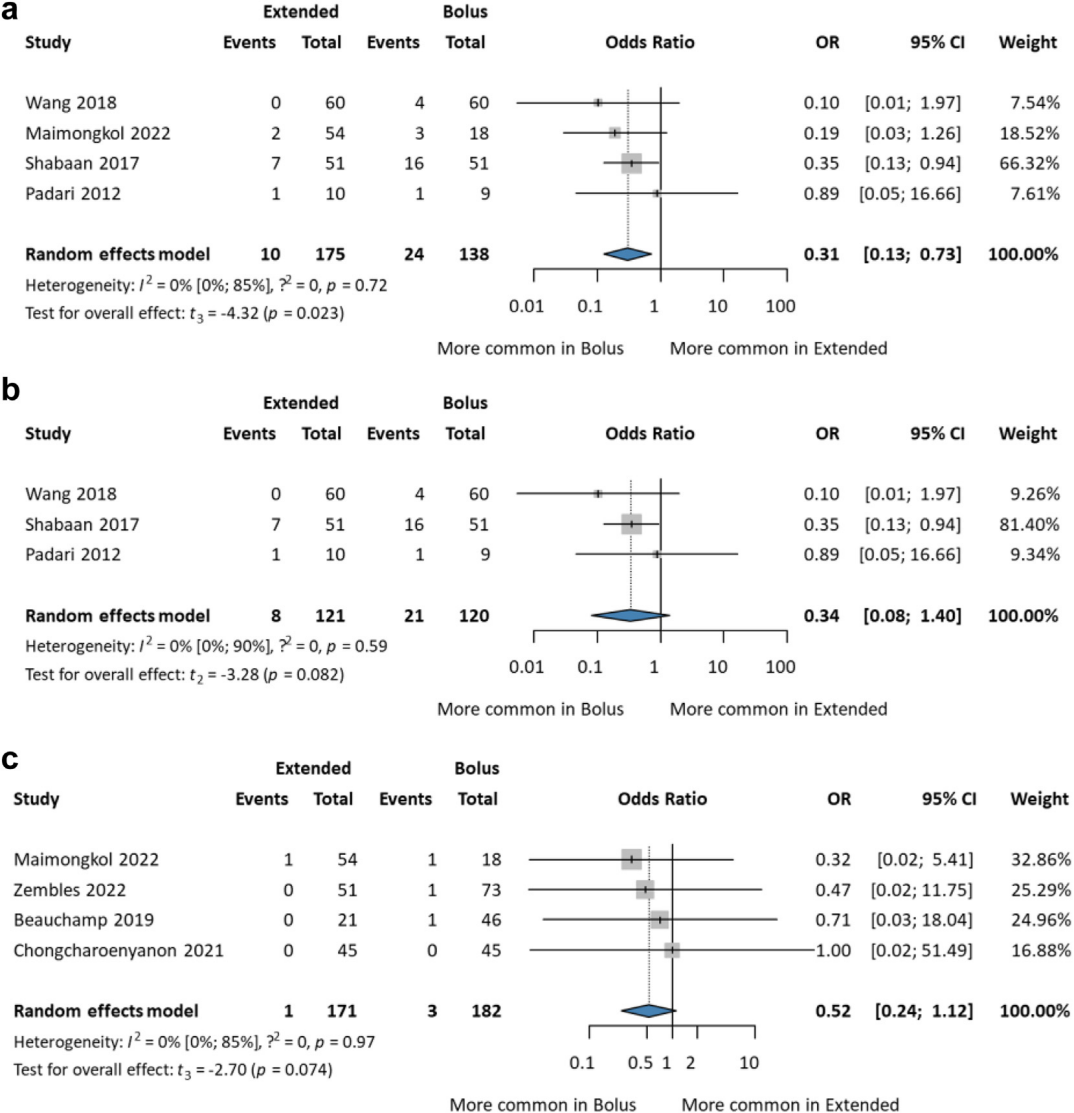
Forest plots of mortality among (a) meropenem treated paediatric patients (b) neonates treated in neonatal intensive care unit (NICU) and (c) infection-related mortality among paediatric patient with a confirmed multidrug-resistant Gram-negative bacteremia (MDR-GNB).

In meropenem-treated patients, mortality in the SI group was significantly (three times) higher than in the EI group [OR 0.31; CI 0.13–0.73]. In the neonatal subset, the result was similar, but non-significant [OR 0.34; CI 0.08–1.40]. However, the study and sample sizes were small (Fig. 3a and b).

Infection-related mortality among children with a confirmed GNB showed EI was more favourable, but the difference was not statistically significant [1/171 vs. 3/182; OR 0.52; CI 0.24–1.12; I^2^ = 0%; CI 0–85%]; and the number of patients was small (Fig. 3c).

### Secondary outcomes

#### No change in clinical cure

**Fig. 4:**
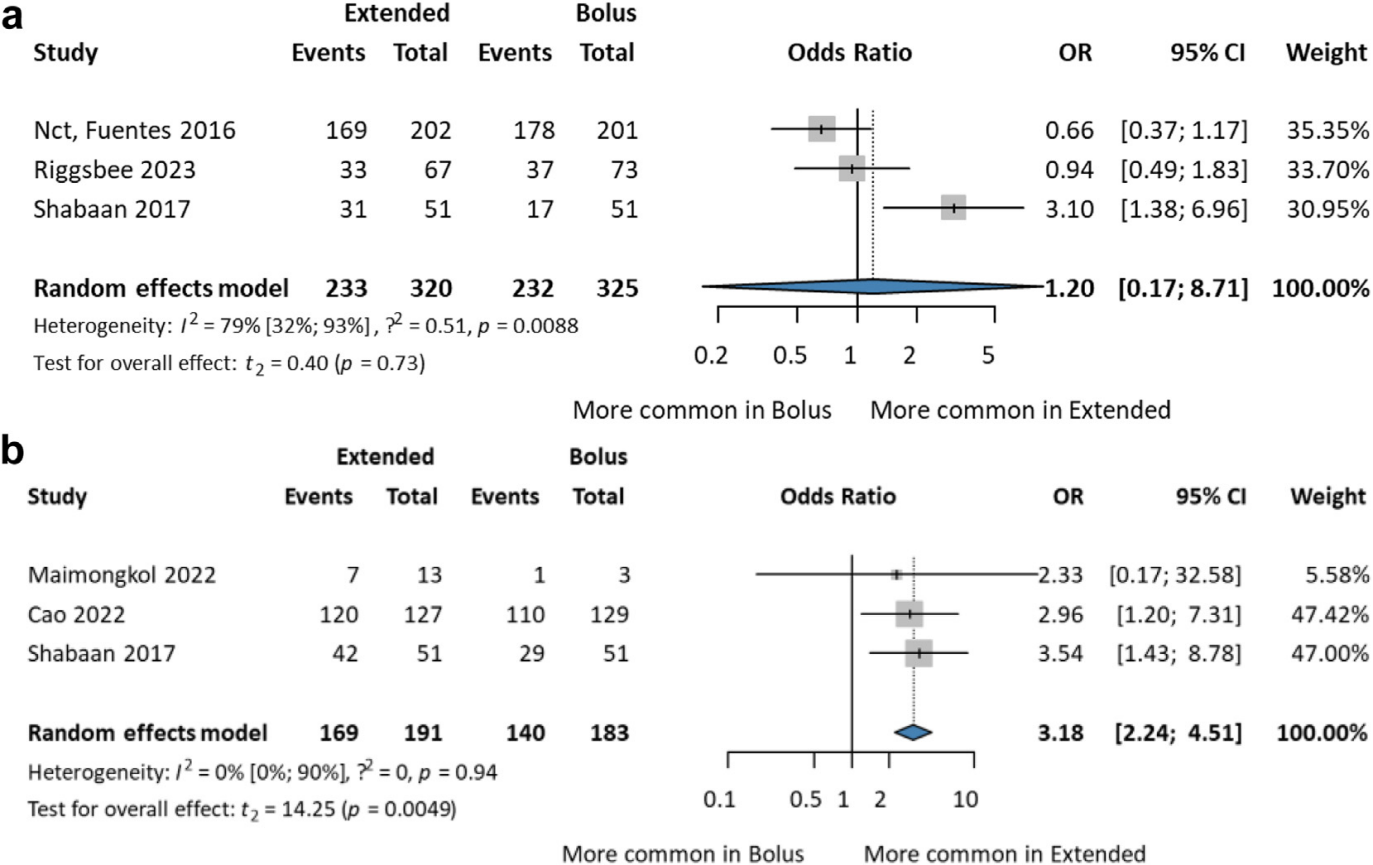
(a) Forest plot of clinical cure, (b) forest plot of microbiological eradication among paediatric patients treated with extended vs. bolus infusion of beta-lactams.

The clinical cure reported complete symptomatic resolution of clinical signs (absence of fever, white blood cell count normalisation, negative follow-up cultures, hemodynamic stability, normal arterial blood gas test values, temperature stability, tolerance for enteral feeding, and discontinuation of inotropes for at least 48-h; CF patients within 5% of baseline ppFEV1 [percent predicted forced expiratory volume] at discharge) related to the infection at the end of therapy or at discharge. No significant difference was found between the two groups [OR 1.20; CI 0.17–8.71; I^2^ = 79%; CI 32–93%]. The sample sizes were small, and the groups were heterogeneous (Fig. 4a). Treatment failure on the third day did not differ significantly between the two groups [OR 0.84; CI 0.34–2.05] (Supplementary Figure 3S).

#### Improvement in microbiological eradication

Microbiological eradication was determined by repeated culture 3–7 days after treatment initiation. All patients were treated with meropenem. In a study by Maimongkol et al., the dose was higher in the EI group than in the SI group.^24^ Significantly, extended infusion was three times more likely to kill bacteria [OR 3.18; CI 2.24–4.51; I^2^ = 0%; CI 0–90%] (Fig. 4b). In a retrospective study the EI of piperacillin/tazobactam resulted in complete microbiologic resolution for all patients, with a 74% (29/39) clinical cure rate at 21 days.^42^

#### Better pharmacokinetic/pharmacodynamic exposure

**Fig. 5:**
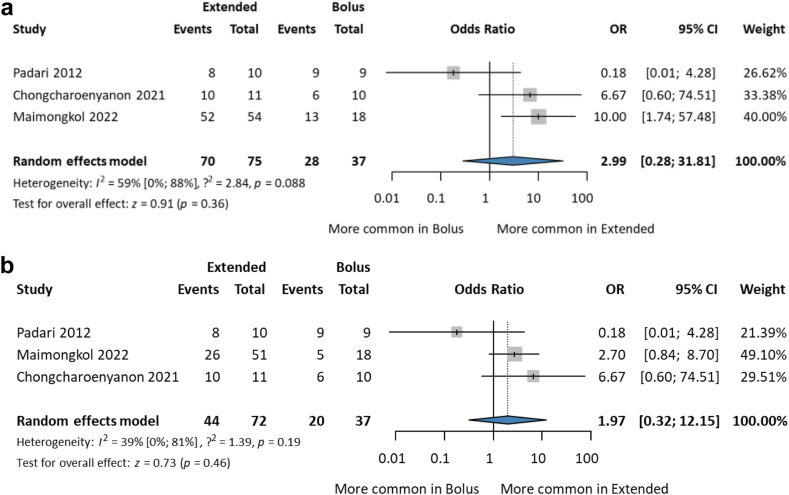
Forest plot of achieving the target 50–100% fT > MIC (a) Maimongkol et al. study used the definition 50% fT > MIC (C_mid_ > MIC, MIC cutoff ≤2 mg/L) (b) Maimongkol et al. study used the definition 100% fT > MIC.

In the study by Maimongkol et al.,^24^ two definitions were used: 50% Ft > MIC (C_mid_ > MIC, MIC cutoff ≤2 mg/L) (Fig. 5a), and 100% fT > MIC (C_trough_ > MIC, MIC cutoff ≤2 mg/L) (Fig. 5b). The extended group might be favourable, but the CI is wide [OR 2.99; CI 0.28–31.81; I^2^% = 59%; CI 0–88%; and OR 1.97; CI 0.32–12.15; I^2^% = 59%; CI 0–81%].

Higher optimal exposure (50–100% fT > 4 × MIC) could not be evaluated, but according to two studies,^3^^,^^22^ EI might be more likely to reach it. In one study, 80 PICU patients received 106 β-lactam courses, and CI provided more optimal PK target (100% fT > 4 × MIC) than SI (n = 22/32, 69% for CI vs. n = 35/74, 47% for SI), together with less underexposure and more overexposure.^40^ Comparing the trough levels of meropenem and piperacillin in paediatric haematology-oncology patients, only 8% of piperacillin levels given continuously were insufficient (out of the range of 30–60 mg/L); however, 67% of SI plasma concentrations showed underexposure (lower than the targets: meropenem 2–8 mg/L, piperacillin 8–30 mg/L).^39^ In a retrospective study more samples reached the target concentration with continuous infusion than with SI (62.2% vs. 38.4%, p < 0.001), and less sample were over- or underdosed, but the target was not defined.^43^

A study among critically ill children (21 patients, 46 measurements) treated with continuous infusion of beta-lactams showed that the optimal target (100% fT > 4 × MIC) was achieved in 76.2% of cases.^41^ Among 11 neonates receiving amoxicillin continuously, only 3 of 22 samples did not reach the PK index of 100% fT > 4 × MIC of *Escherichia coli*.^44^

#### No difference in acute kidney injury (AKI)

**Fig. 6:**
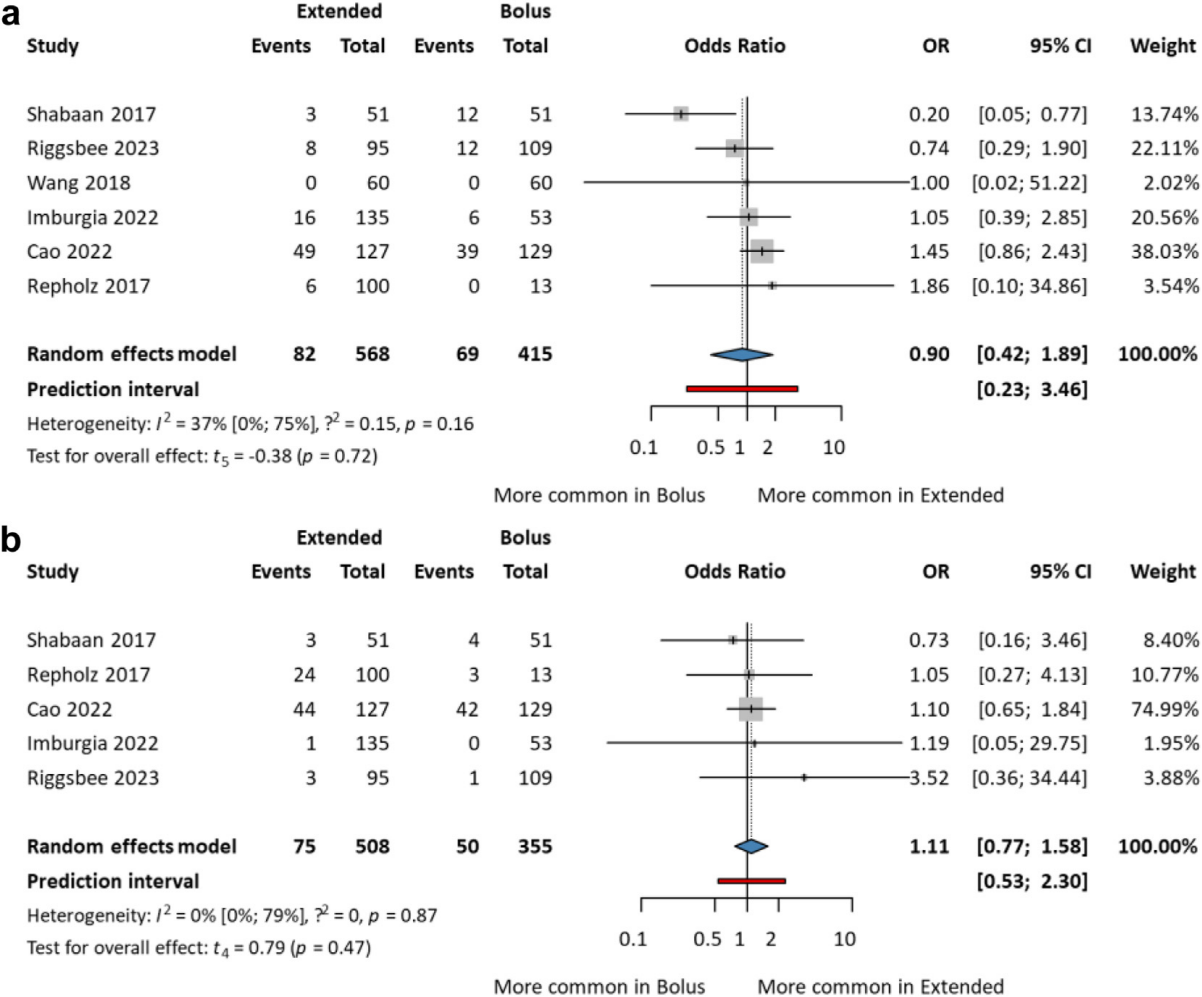
Forest plots of adverse events among paediatric patients treated with extended vs. bolus infusion of beta-lactams (a) acute kidney injury (b) hepatic adverse events.

AKI was defined as either an elevated creatinine level or by using KDIGO guidelines. These studies included neonates and children with CF. Alterations in AKI events were not statistically significant [OR 0.90; CI 0.42–1.89; I^2^ = 37%; CI 0–75%] (Fig. 6a). There was also no statistically significant difference between CF patients and neonates [OR 0.90; CI 0.42–1.89] (Supplementary Figure 4S).

#### No difference in hepatic adverse events

Hepatic adverse events included any abnormalities in transaminase levels, and regarding study by Riggsbee et al. we chose ALT levels to identify hepatotoxicity.^38^ Similarly to AKI, there was no statistically significant difference between the two groups [OR 1.11; CI 0.77–1.58; I^2^ = 0%; CI 0–79%] (Fig. 6b). Both types of administrations were considered safe. However, the investigation of all adverse events was difficult, and the interpretation can be problematic because of the paucity and variability of the definitions (Supplementary Figure 5S).

#### No difference in LOS, PICU length of stay and duration of the antibiotic

LOS, PICU length of stay, and duration of antibiotic course were expressed in days. No significant differences were found [LOS: OR 0.90; CI 0.52–2.32] (Supplementary Figure 6S).

### Risk of bias assessment

The overall risk of bias was low among RCTs. However, we assessed some concerns in the study by Chongcharoenyanon et al.^22^ due to baseline differences between intervention groups, missing data, and selection of the reported result, and in the study by Wang et al.^21^ due to the selection of the reported result. The overall risk of bias was high among non-RCTs, mainly due to confounding factors, inaccurate selection of participants, and missing data. The quality of evidence ranged between very low to moderate because of the small sample size, overall high risk of bias, and clinically heterogeneous populations.

The summary figures of the risk of bias assessment and the summary of the findings table for GRADE are provided in the Supplementary Material (Supplementary Figures 10S and 11S and Supplementary Table 5S).

## Discussion

Overall, patients died approximately 26% lower odds [CI 0.55; 0.9] in the EI group than in the SI group. The total mortality of the studies ranged 0–35.8% and was very high among patients with meningitis (>35%).^28^^,^^29^

Previous meta-analyses of adults have shown the advantages of EI in sepsis and respiratory infections.^8^^,^^9^^,^^45^ In a meta-analysis of RCTs, a non-significant association was found between EI and lower mortality; however, one study showed the advantage of EI with ceftazidime in critically ill adults with severe infections (EI: 3/10, SI: 9/11 deaths).^46^ In a study among critical care paediatric patients, those who received EI had a lower all-cause mortality rate within 30 days of completing antibiotics compared to SI (2.1% vs. 19.6%; p = 0.006).^32^ Mortality is influenced by comorbidities, the presence of immune deficiency, the need for respiratory support, and the need for inotropic therapy. The Acute Physiology and Chronic Health Evaluation (APACHE) II score (ranging 0–71) is a severity index with higher scores indicating an increased risk of death. In an analysis of individual patient data, mortality rates were slightly lower in the continuous infusion group for patients with an APACHE II score of ≥22 [RR 0.74; 95% CI 0.53–1.01], but not for those with a score <22 [RR 0.69; 95% CI 0.39–1.21].^47^ EI may offer greater benefit for critically ill patients with severe infections.

The subset analysis of all-cause mortality among meropenem-treated patients found similar results to those of previous meta-analyses in a severely ill population: fatalities in the EI group were significantly lower than those in the SI group.^48^^,^^49^ In the literature, no significant differences have been found between beta-lactam subclasses.^8^^,^^45^^,^^46^

The occurrence of proven bacteraemia is low or unknown, and the relevance of all-cause mortality is debatable.^25^ Examining infection-related mortality, deaths occurred nearly 50% less likely in the EI group, which is a clinically relevant, but not significant result [CI 0.24; 1.12]. An adult RCT reported ventilator-associated pneumonia (VAP)-related mortality, and the extended group (amikacin + EI meropenem + nebulized amikacin; 4/30) was associated with lower mortality than the control group (amikacin + SI meropenem 8/30).^50^ However, the additional nebulized antibiotic might have also contributed to the reduction.

In contrast to mortality, no differences were found in clinical cure or treatment failure. However, two neonatal studies have shown the clinical effectiveness of EI.^33^^,^^34^ In another newborn study, the duration of clinical symptoms remission was shorter in the extended group (3.1 ± 1.8 days vs. 6.2 ± 1.6 days, p = 0.036).^21^ The EI of meropenem may be beneficial for late-onset neonatal sepsis. In previous meta-analyses, meropenem EI had a significantly higher cure rate than SI.^48^^,^^49^

The clinical cure reported in previous adult meta-analyses among adults showed that EI is slightly more favourable than SI, however there were discrepancies between clinical cure and mortality due to the subjectivity and heterogeneity of its definition.^8^^,^^9^^,^^45^, ^46^, ^47^ EI was preferred for more seriously ill patients with pneumonia; the clinical cure rate was higher in patients with APACHE II scores ≥15 receiving EI vs. SI.^45^

Microbiological eradication was the most objective measure of efficacy. Only three meropenem studies have reported pathogenic culture data, demonstrating the effectiveness of EI.^51^ Two meta-analyses that administered meropenem in severe infections showed a significantly higher bacterial eradication rate in the EI group.^48^^,^^49^

Appropriate plasma concentrations are crucial for eradication. Critically ill children may be exposed to insufficient antibiotic levels due to alterations in PK properties (such as elevated glomerular filtration rate), fluid shifts, systematic inflammation response and treatment with vasoactive agents.^5^^,^^6^ According to a study carried out in a PICU, augmented renal clearance (ARC, creatinine clearance CrCl ≥130 mL/min/1.73 m^2^) was present for at least one day in 62 of 92 (67.4%) patients.^52^ The level of antimicrobial exposure was inadequate in 85% of patients with ARC in an included study.^39^ Plasma meropenem concentrations were lower in patients with ARC than without ARC in another study.^24^

Based on paediatric PK studies, which employed Monte Carlo simulations to determine the effects of modifying components of an antimicrobial drug regimen, EI improved the exposure compared to SI.^2^ However, plasma concentration measurements were limited to only seven comparative studies,^3^^,^^22^^,^^24^^,^^35^^,^^39^, ^40^, ^43^ and only three could be included in the meta-analysis. Therapeutic drug monitoring (TDM)-guided dosing enhanced the clinical and microbiological cure and treatment response in critically ill adults but did not affect the mortality. The inclusion of non-septic patients could distort the mortality data.^51^ Similarly, we also included patients with suspected infection without detection of bacteria. Patients with optimal target (100% Ft > 4 × MIC) had both a significantly higher microbiological eradication rate and lower resistance development rate than underexposed patients in a study.^41^ However, we could not evaluate the association between the target achievement and the efficacy.

No serious adverse events were reported, and there was no difference in AKI and hepatotoxicity between the groups, which was consistent with previous adult experiences.^8^^,^^45^^,^^46^^,^^48^ Concerning cost effectiveness no difference is expected due to the same daily dose and the non-significant difference in the length of stay and duration of the antibiotic course.

Technical issues such as intravenous (IV) access concerns, IV compatibility and antibiotic stability are important in the implementation of extended or continuous infusion in practice. In a study EI was initiated in 143 patients, and the infusion time was changed only in 11 patients. In 6 cases there were IV access issues (incompatibility/not enough access, preventive change, patient freedom, fear of precipitation).^27^ When changing the infusion, stability must be considered, for example meropenem is stable for 3 h after dissolution in room temperature according to the monographs, and continuous infusion can be problematic.

Regarding the strengths of our study, we followed our protocol (only infection-related mortality was an additional outcome), which was registered in PROSPERO in advance. Rigorous methodology was applied.

Our meta-analysis has several limitations. First, a small number of cases were enrolled in some outcomes, and RCTs and observational cohorts were analysed together. Second, wide range of patients were involved according to age and comorbidities. Third, the posology differed depending on whether a bolus infusion was administered before the first prolonged infusion. Fourth, in some cases, the pathogen was only suspected, or microbiological culture data were not available. Fifth, concomitant antibiotics were used in 10 studies, or sometimes were not reported. Sixth, the follow-up duration was inconsistent in the cases of mortality. Seventh, the definitions of clinical cure differed among studies, and the assessment was subjective. Eighth, there were missing data in TDM, target attainment and microbiological eradication. Ninth, creatinine abnormalities might not directly translate to kidney injury, and this number can be overestimated in preterm neonates. Tenth, the non-RCTs had a high risk of bias. Eleventh, publication bias could not be assessed correctly because of the small number of included studies.

Based on our results, extended or continuous infusion of beta-lactams should be considered, especially for neonates and critically ill children, and among meropenem-treated patients. Therapeutic drug monitoring is necessary.

Further prospective data collection (comparative clinical trials) with plasma concentration measurements is needed to assess the problem in question more accurately.^53^

In conclusion, extended infusion of beta-lactams showed a significantly lower all-cause mortality rate and earlier microbiological eradication in children. No higher risk of toxicity was identified, the extended infusion seems to be as safe as the short-term infusion.

## Contributors

KAB: conceptualisation, investigation, project administration, methodology, formal analysis, accessing and verifying the data, writing—original draft; MO, VM: conceptualisation, methodology, project administration, visualization, validation, writing—review & editing; RN, PH, MG, BH: conceptualisation, writing—review & editing; ÁET: conceptualisation, investigation, writing—review & editing; AH, SK-D: conceptualisation, formal analysis, software, writing—review & editing; CL: conceptualisation, supervision, validation, accessing and verifying the data, writing—original draft.

All authors certify that they have participated sufficiently in the work to take public responsibility for the content, including participation in the concept, design, analysis, writing, or revision of the manuscript. All authors agree with the final version of the manuscript.

## Data sharing statement

All extracted data supporting the findings of this specific systematic review and meta-analysis are available upon request after approval of a proposal from the corresponding author (CL, lodi.csaba@med.semmelweis-univ.hu).

## Declaration of interests

All authors declare no competing interests.
