# Supplementary material for: Extended infusion of β-lactams significantly reduces mortality and enhances microbiological eradication in paediatric patients: a systematic review and meta-analysis
Source: eClinicalMedicine. 2023 Nov 2;65:102293. doi: 10.1016/j.eclinm.2023.102293 (PMC10651452; doi:10.1016/j.eclinm.2023.102293)
Supplement: Supplementary Material [file mmc1.docx]

**Supplementary material**

**Title**

**Extended Infusion of β-lactams Significantly Reduces Mortality and Enhances Microbiological Eradication in Paediatric Patients:** A Systematic Review and Meta-Analysis

**Authors**

Kinga Anna Budai^a,b^, Ágnes Eszter Tímár^b,c^, Mahmoud Obeidat^b^, Vanda Máté^b,d^, Rita Nagy^b,c,e^, Andrea Harnos^b,f^, Szilvia Kiss-Dala^b^, Péter Hegyi^b,e,g^, Miklós Garami^b,d^, Balázs Hankó^a,b^, Csaba Lódi^b,d*^, lodi.csaba@med.semmelweis-univ.hu

**Affiliations:**

1. University Pharmacy, Department of Pharmacy Administration, Semmelweis University, Budapest, Hungary
2. Centre for Translational Medicine, Semmelweis University, Budapest, Hungary
3. Heim Pál National Pediatric Institute, Budapest, Hungary
4. Pediatric Center, MTA Center of Excellence, Semmelweis University, Budapest, Hungary
5. Institute for Translational Medicine, Medical School, University of Pécs, Pécs, Hungary
6. Department of Biostatistics at the University of Veterinary Medicine, Budapest, Hungary
7. Institute of Pancreatic Diseases, Semmelweis University, Budapest, Hungary

***Corresponding author**

Pediatric Center, MTA Center of Excellence, Semmelweis University, Bókay János u. 53-54. Budapest, H-1083 Hungary

**Table of contents:**

Table 1S. Search key (Page 3)

Detailed description of synthesis methods (Page 3)

Table 2S. List of studies excluded at full-text screening stage (Page 4)

The hypotheses of the RCTs for calculating the number of patient (Page 9)

Figure 1S. Forest plots of all-cause mortality among paediatric patients treated with extended versus bolus infusion of beta-lactams, including RCT. (a) all studies (b) without meningitis studies (Page 9)

Figure 2S. Forest plot of all-cause mortality among paediatric patients treated with extended versus bolus infusion of beta-lactams, including non-RCTs (Page 10)

Figure 3S. Forest plot of treatment failure on the 3rd day among paediatric patients treated with extended versus bolus infusion of beta-lactams (Page 10)

Figure 4S. Forest plots of acute kidney injury (AKI), subgroup analysis of cystic fibrosis (CF) patients and neonates (Page 11)

Figure 5S. Forest plots of all adverse events among paediatric patients treated with extended versus bolus infusion of beta-lactams, subgroup analysis of RCTs and non-RCTs (Page 12)

Figure 6S. Forest plots of (a) the duration of the antibiotic (b) LOS (c) PICU length of stay among paediatric patients treated with extended versus bolus infusion of beta-lactams (Page 13)

Table 3S. Raw data table for the duration of antibiotic course (Page 14)

Table 4S. Raw data table for the length of hospital stay (Page 14)

Figure 7S. Funnel plots of mortality among paediatric patients treated with extended versus bolus infusion of beta-lactams. (a) all studies (b) without meningitis studies (Page 15)

Figure 8S. Influential analyses of studies with all-cause mortality outcome. (a) all studies (b) without meningitis studies (Page 17)

Figure 9S. Leave-one-out analyses, sorted by effect size plots of studies with all-cause mortality outcome. (a) all studies (b) without meningitis studies (Page 19)

Figure 10S. Risk of bias assessment of each outcome using the revised Cochrane risk-of-bias tool for RCTs (RoB2) (Page 21)

Figure 11S. Risk of bias assessment of each outcome using the revised Cochrane risk-of-bias tool non-RCTs (ROBINS-I) (Page 22)

Table 5S. Summary of findings: Grading of Recommendations, Assessment, Development and Evaluations (GRADE) framework (Page 23)

**Table 1S. Search key in the databases (MEDLINE, Embase, CENTRAL, and Scopus)**

| (pediatric* OR paediatric* OR adolescent OR adolescence OR child* OR "young adult" OR "young adults" OR kids OR kid OR youth OR juvenile OR infant* OR infancy OR preschooler* OR teen OR teens OR teenager* OR neonat* OR premat* OR newborn) AND |
| --- |
| (intermittent OR bolus OR short OR extended OR extended OR continuous OR discontinuous) AND |
| (duration OR infusion OR administration OR interval OR dosing) AND |
| (β-lactam* OR beta-lactam* OR carbapenem OR cephalosporin OR monobactam OR penicillin OR benzylpenicillin OR dicloxacillin OR flucloxacillin OR amoxicillin OR ampicillin OR piperacillin OR tazobactam OR cefazolin OR cefuroxime OR ceftazidime OR ceftriaxone OR cefixime OR cefalotin OR cefotaxime OR cefepime OR ceftaroline OR doripenem OR ertapenem OR meropenem OR imipenem OR cilastatin OR aztreonam) |

**Detailed description of synthesis methods**

We provide the following additional details on data synthesis.

For pooling OR, the Mantel-Haenszel method^1,2^ was performed. Exact Mantel-Haenszel method (without continuity correction) was used to handle zero cell counts (as recommended^3,4^). For calculating the CI of the (estimated) difference between group medians, the quantile estimation method was used. In the case of the length of hospital stay outcome, the quantile estimation in each study was based on the best fitting normal, log-normal, gamma, Weibull distribution as referred S2 scenario in McGrath’s article^5^. Regarding the duration of the antibiotic course, two studies^6,7^ reported the mean and the standard deviation while all the others reported the median and the quantiles, so for these two studies the median and quantile values were estimated assuming a normal distribution. Based on the other publications in the literature about this outcome and the data in the used publication, we could assume that the distribution of this variable is not relevantly different from a normal distribution. Inverse variance weighting method was used to calculate the pooled difference of medians. According to the article by McGrath et al.^5^, when some studies report the mean and others report the median, it is more advantageous to pool median values.

We used a Hartung-Knapp adjustment^8,9^ for CIs. To estimate the heterogeneity variance measure (τ^2), for OR calculation the Paule-Mandel method^10^ (recommended by Veroniki et al.^11^) was used. Prediction interval calculations were based on the t-distribution. For difference of medians, the restricted maximum-likelihood estimator was used with the Q profile method for confidence interval.

On the forest plots in case of 0 cell counts, individual study OR with 95% CI was calculated by adding 0.5 as continuity correction (it was used only for visualization on forest plot).

In the case of subgroup analysis, we used a fixed-effects “plural” model (aka. mixed-effects model). We assumed that all subgroups share a different τ^2 value. To assess the difference between the subgroups a “Cochrane Q” test (an omnibus test) was used between subgroups^12^.

The subgroup analysis was planned before the data extraction in the case of individual antibiotics in the beta-lactam class, neonates and patients between 1 month - 21 years, different patient characteristics (diseases/infections) variables.

In case of study design (RCTs and non-RCTs) variable, the subgroup analysis was decided after data extraction as the data implied this structure.

Funnel plots, influential analyses and leave-one-out plots are included only for the all-cause mortality outcomes, as in the other cases the number of studies was low therefore, they have a limited diagnostic value. We performed the following influential analyses.

- **Plots with leave-one-out analysis values**:

*In leave-on-out analysis we calculate the parameters of interest excluding the studies one-by-one. If a study is an outlier regarding the calculated parameter, it is shown with red color.*

The assessed parameters are:

- Θ: effect size: *the pooled effect* size while omitting one study at a time.
- 95% CI: *the 95% confidence interval* of the pooled effect size while omitting one study at a time.
- I^2^: *the Higgins&Thomson* $I^{2}$ *heterogeneity* value while omitting one study at a time.
- Stand. residual: *the studentized residuals*. It shows the deleted residual divided by its estimated standard deviation.
- Dffits: *the difference in fits*. It quantifies the number of standard deviations that the fitted value changes while omitting one study at a time. (Typical threshold is $3*\sqrt{\frac{p}{k-p}}$, where p is the number of model coefficients and k the number of cases)
- Cook’s Distance: *Cook’s distance*. It depends on both the residual and leverage of the omitted study. (Typical threshold value is 2)
- Covariance Ratio: *the covariance ratio*. It shows the change in the determinant of the covariance matrix of the effect size. (Typical threshold value is 1)
- Tau-squared: *the square of tau value* (random effects variance, between study heterogeneity). Tau-squared value while omitting one study at a time.
- Q: *test statistics of tau-squared*. Q value while omitting one study at a time.
- Hat value: *the value of the hat matrix* while omitting one study at a time. (Typical threshold is $3*\frac{p}{k}$)
- weight: *study weight* in the analysis.

**Table 2S. List of studies excluded at full-text screening stage.**

|  | **References** | **Exclusion reason from the meta-analysis** | **Included in the systematic review** |
| --- | --- | --- | --- |
| 1 | Aardema H, Nannan Panday P, Wessels M, et al. Target attainment with continuous dosing of piperacillin/tazobactam in critical illness: a prospective observational study. Int J Antimicrob Agents. 2017;50(1):68-73. doi:10.1016/j.ijantimicag.2017.02.020 | not the target population | no |
| 2 | André P, Diezi L, Dao K, et al. Ensuring Sufficient Trough Plasma Concentrations for Broad-Spectrum Beta-Lactam Antibiotics in Children With Malignancies: Beware of Augmented Renal Clearance!. Front Pediatr. 2022;9:768438. Published 2022 Jan 5. doi:10.3389/fped.2021.768438 | the outcome (optimal exposure with targeted concentration range) could not be pooled with others | yes |
| 3 | Bakker W, Vinks AA, Mouton JW, de Jonge P, Verzijl JG, Heijerman HG. Continue intraveneuze thuisbehandeling van luchtweginfecties met ceftazidim via een draagbare pomp bij patiënten met cystische fibrose; een multicentrisch onderzoek [Continuous intravenous home treatment of airway infections using ceftazidime administration via portable pump in patients with cystic fibrosis; a multicenter study]. Ned Tijdschr Geneeskd. 1993;137(48):2486-2491. | not the target population | no |
| 4 | Bao H, Lv Y, Wang D, Xue J, Yan Z. Clinical outcomes of extended versus intermittent administration of piperacillin/tazobactam for the treatment of hospital-acquired pneumonia: a randomized controlled trial. Eur J Clin Microbiol Infect Dis. 2017;36(3):459-466. doi:10.1007/s10096-016-2819-1 | not the target population | no |
| 5 | Bates A, Joffe AR. Is there a role for continuous infusion of β-lactam antibiotics in severe sepsis?. J Thorac Dis. 2016;8(6):E437-E439. doi:10.21037/jtd.2016.03.81 | wrong publication type | no |
| 6 | Battersby NC, Patel L, David TJ. Increasing dose regimen in children with reactions to ceftazidime. Clin Exp Allergy. 1995;25(12):1211-1217. doi:10.1111/j.1365-2222.1995.tb03045.x | the outcomes (adverse events) could not be pooled with others | no |
| 7 | Benech N, Dumitrescu O, Conrad A, et al. Parameters influencing the pharmacokinetics/pharmacodynamics of piperacillin/tazobactam in patients with febrile neutropenia and haematological malignancy: a prospective study. J Antimicrob Chemother. 2019;74(9):2676-2680. doi:10.1093/jac/dkz248 | not the target population | no |
| 8 | Beringer P, Shapiro B, Han E, Louie S, Gill M, Rao A. Pharmacodynamics (PD) of continuous infusion (CI) cefepime in adult cystic fibrosis (CF) patients. Pediatric pulmonology, 2003, Suppl 25, 297‐298 | not the target population | no |
| 9 | Bertels RA, Semmekrot BA, Gerrits GP, Mouton JW. Serum concentrations of cefotaxime and its metabolite desacetyl-cefotaxime in infants and children during continuous infusion. Infection. 2008;36(5):415-420. doi:10.1007/s15010-008-7274-1 | no comparator | no |
| 10 | Bosso JA, Bonapace CR, Flume PA, White RL. A pilot study of the efficacy of constant-infusion ceftazidime in the treatment of endobronchial infections in adults with cystic fibrosis. Pharmacotherapy. 1999;19(5):620-626. doi:10.1592/phco.19.8.620.31525 | not the target population | no |
| 11 | Breuer O, Cohen-Cymberknoh M, Armoni S, Kerem E, Shoseyov D. Continuous intravenous β-lactam antibiotics in cystic fibrosis patients with severe drug hypersensitivity. Ann Allergy Asthma Immunol. 2014;113(2):229-230. doi:10.1016/j.anai.2014.05.014 | not the target population | no |
| 12 | Byl B, Baran D, Jacobs F, Herschuelz A, Thys JP. Serum pharmacokinetics and sputum penetration of amikacin 30 mg/kg once daily and of ceftazidime 200 mg/kg/day as a continuous infusion in cystic fibrosis patients. J Antimicrob Chemother. 2001;48(2):325-327. doi:10.1093/jac/48.2.325  (duplicated) | not the target population | no |
| 13 | Carrié C, Petit L, d'Houdain N, et al. Association between augmented renal clearance, antibiotic exposure and clinical outcome in critically ill septic patients receiving high doses of β-lactams administered by continuous infusion: a prospective observational study. Int J Antimicrob Agents. 2018;51(3):443-449. doi:10.1016/j.ijantimicag.2017.11.013 | not the target population | no |
| 14 | Cies JJ, Moore WS 2nd, Enache A, Chopra A. β-lactam Therapeutic Drug Management in the PICU. Crit Care Med. 2018;46(2):272-279. doi:10.1097/CCM.0000000000002817 | the outcomes could not be pooled with others (there were overall outcomes, not group specific) | no |
| 15 | Cojutti PG, Lazzarotto D, Candoni A, et al. Real-time TDM-based optimization of continuous-infusion meropenem for improving treatment outcome of febrile neutropenia in oncohaematological patients: results from a prospective, monocentric, interventional study. J Antimicrob Chemother. 2020;75(10):3029-3037. doi:10.1093/jac/dkaa267 | not the target population | no |
| 16 | Colding H, Andersen GE. Administration of gentamicin and ampicillin by continuous intravenous infusion to newborn infants during parenteral nutrition. Scand J Infect Dis. 1982;14(1):61-65. doi:10.3109/inf.1982.14.issue-1.12 | wrong study design | no |
| 17 | Colding H, Møller S, Andersen GE. Continuous intravenous infusion of ampicillin and gentamicin during parenteral nutrition in 88 newborn infants. Arch Dis Child. 1982;57(8):602-606. doi:10.1136/adc.57.8.602 | wrong study design | no |
| 18 | Cotrina-Luque J, Gil-Navarro MV, Acosta-García H, et al. Continuous versus intermittent piperacillin/tazobactam infusion in infection due to or suspected pseudomonas aeruginosa. Int J Clin Pharm. 2016;38(1):70-79. doi:10.1007/s11096-015-0208-y | not the target population | no |
| 19 | Dalle JH, Gnansounou M, Husson MO, Lambilliotte A, Mazingue F, Nelken B. Continuous infusion of ceftazidime in the empiric treatment of febrile neutropenic children with cancer. J Pediatr Hematol Oncol. 2002;24(9):714-716. doi:10.1097/00043426-200212000-00006 | no comparator | no |
| 20 | David T.J., Devlin J. Continuous infusion of ceftazidime in cystic fibrosis. Lancet 1989 1:8652 (1454-1455). | the outcomes (mean serum drug levels) could not be pooled with others | no |
| 21 | Debray, A., Callot, D., Hirt, D. et al. Beta-lactam exposure and safety in intermittent or continuous infusion in critically ill children: an observational monocenter study. Eur J Pediatr 182, 965–973 (2023). https://doi.org/10.1007/s00431-022-04716-0 | the outcomes could not be pooled with others (data based on antibiotic courses, not patients) | yes |
| 22 | de Cacqueray N, Boujaafar S, Bille E, et al. Therapeutic Drug Monitoring of Antibiotics in Critically Ill Children: An Observational Study in a Pediatric Intensive Care Unit. Ther Drug Monit. 2022;44(2):319-327. doi:10.1097/FTD.0000000000000918 | wrong drug and dosing | no |
| 23 | De Keukeleire S, Borrey D, Decaluwe W, Reynders M. Therapeutic Drug Monitoring of Meropenem in Neonate with Necrotizing Enterocolitis: A Challenge. Case Rep Infect Dis. 2016;2016:6207487. doi:10.1155/2016/6207487 | wrong publication type | no |
| 24 | Draime J., Nicholls J., Sibbitt B., Gryka R., Simpson D. A comparison of the effect of short intermittent and prolonged intermittent infusion of meropenem on the prevalence of nausea in pediatric patients with cystic fibrosis.  J. Am. Pharm. Assoc. 2014;54(2):e211. doi: 10.1331/JAPhA.2014.14511 | wrong outcome | no |
| 25 | Egerer G., Goldschmidt H., Salwender, H. et al. Efficacy of continuous infusion of ceftazidime for patients with neutropenic fever after high-dose chemotherapy and peripheral blood stem cell transplantation. Int. J. Antimicrob. Agents. 2000;15(2):119-123. doi: 10.1016/S0924-8579(00)00155-2 | not the target population | no |
| 26 | Meropenem pharmacokinetics in very low birth weight neonates. https://trialsearch.who.int Indentifier: EUCTR2009‐017823‐24‐EE. | wrong publication type | no |
| 27 | Fangel S., Fuursted K., Olesen H.V., Norskov N., Schiøtz P.O., Petersen E. Treatment of cystic fibrosis patients with continuous infusion of antibiotics. J. Cyst. Fibrosis. 2010 9 (S39) SUPPL. 1 | wrong publication type | no |
| 28 | Gatti M, Campoli C, Latrofa ME, et al. Relationship Between Real-time TDM-guided Pharmacodynamic Target Attainment of Continuous Infusion Beta-lactam Monotherapy and Microbiologic Outcome in the Treatment of Critically Ill Children With Severe Documented Gram-negative Infections [published online ahead of print, 2023 Jul 24]. Pediatr Infect Dis J. 2023;10.1097/INF.0000000000004054. doi:10.1097/INF.0000000000004054 | no comparator | yes |
| 29 | Giacchino M, Bezzio S, Chiapello N, et al. Continuous antibiotic infusion for salvage therapy of partially implanted central venous catheter tunnel infections due to staphylococci. Pediatr Blood Cancer. 2007;49(7):1010-1012. doi:10.1002/pbc.20864 | wrong drug | no |
| 30 | Hanes SD, Wood GC, Herring V, et al. Intermittent and continuous ceftazidime infusion for critically ill trauma patients. Am J Surg. 2000;179(6):436-440. doi:10.1016/s0002-9610(00)00388-3 | not the target population | no |
| 31 | Hong L.T., Bhakta Z.N., Stevens V. et al. Pharmacokinetics of continuous infusion beta-lactams in the treatment of acute pulmonary exacerbations in adult cystic fibrosis patient. Pediatr. Pulmonol. 2015 50 (321) SUPPL. 41 doi:10.1002/ppul.23297 | not the target population | no |
| 32 | Hubert D, Le Roux E, Lavrut T, et al. Continuous versus intermittent infusions of ceftazidime for treating exacerbation of cystic fibrosis. Antimicrob Agents Chemother. 2009;53(9):3650-3656. doi:10.1128/AAC.00174-09 | not the target population | no |
| 33 | Hubert D, Wallaert B, Scheid P et al. Continuous infusion versus intermittent administration of ceftazidime in cystic fibrosis patients. Pediatric pulmonology, 2003, Suppl 25, 294 | not the target population | no |
| 34 | Hughes DW, Frei CR, Maxwell PR, et al. Continuous versus intermittent infusion of oxacillin for treatment of infective endocarditis caused by methicillin-susceptible Staphylococcus aureus. Antimicrob Agents Chemother. 2009;53(5):2014-2019. doi:10.1128/AAC.01232-08 | not the target population | no |
| 35 | Jamal JA, Mat-Nor MB, Mohamad-Nor FS, et al. Pharmacokinetics of meropenem in critically ill patients receiving continuous venovenous haemofiltration: a randomised controlled trial of continuous infusion versus intermittent bolus administration. Int J Antimicrob Agents. 2015;45(1):41-45. doi:10.1016/j.ijantimicag.2014.09.009 | not the target population | no |
| 36 | Jaruratanasirikul S, Limapichat T, Jullangkoon M, Aeinlang N, Ingviya N, Wongpoowarak W. Pharmacodynamics of meropenem in critically ill patients with febrile neutropenia and bacteraemia. Int J Antimicrob Agents. 2011;38(3):231-236. doi:10.1016/j.ijantimicag.2011.04.019 | not the target population | no |
| 37 | Jaruratanasirikul S, Sriwiriyajan S, Punyo J. Comparison of the pharmacodynamics of meropenem in patients with ventilator-associated pneumonia following administration by 3-hour infusion or bolus injection. Antimicrob Agents Chemother. 2005;49(4):1337-1339. doi:10.1128/AAC.49.4.1337-1339.2005 | not the target population | no |
| 38 | Knoderer CA, Karmire LC, Andricopulos KL, Nichols KR. Extended Infusion of Piperacillin/Tazobactam in Children. J Pediatr Pharmacol Ther. 2017;22(3):212-217. doi:10.5863/1551-6776-22.3.212 | no comparator | yes |
| 39 | Kuzemko J, Crawford C. Continuous infusion of ceftazidime in cystic fibrosis. Lancet. 1989;2(8659):385. doi:10.1016/s0140-6736(89)90561-8 | not the target population | no |
| 40 | Lau WK, Mercer D, Itani KM, et al. Randomized, open-label, comparative study of piperacillin-tazobactam administered by continuous infusion versus intermittent infusion for treatment of hospitalized patients with complicated intra-abdominal infection. Antimicrob Agents Chemother. 2006;50(11):3556-3561. doi:10.1128/AAC.00329-06 | not the target population | no |
| 41 | Leder K., Turnidge J.D., Korman T.M., Grayson M.L. The clinical efficacy of continuous-infusion flucloxacillin in serious staphylococcal sepsis. J. Antimicrob. Chemother. 1999;43:1 (113-118) DOI: 10.1093/jac/43.1.113 | not the target population | no |
| 42 | Lipš M, Siller M, Strojil J, Urbánek K, Balík M, Suchánková H. Pharmacokinetics of imipenem in critically ill patients during empirical treatment of nosocomial pneumonia: a comparison of 0.5-h and 3-h infusions. Int J Antimicrob Agents. 2014;44(4):358-362. doi:10.1016/j.ijantimicag.2014.05.011 | not the target population | no |
| 43 | Louie J.M., Young D.C., Zobell J.T. et al. Safety of continuous infusion beta-lactam antibiotics in adult cystic fibrosis patients during an acute pulmonary exacerbation. Pediatr. Pulmonol. 2014 49 (334-335) SUPPL. 38. DOI: 10.1002/ppul.23108 | not the target population type | no |
| 44 | Maarbjerg SF, Thorsted A, Friberg LE, et al. Continuous infusion of piperacillin-tazobactam significantly improves target attainment in children with cancer and fever. Cancer Rep (Hoboken). 2022;5(10):e1585. doi:10.1002/cnr2.1585 | wrong study design | no |
| 45 | Marmo E., Coppola L., Pempinello R., Di Nicuolo G, Lampa E. Levels of amoxycillin in the liquor during continuous intravenous administration. Chemotherapy (1982) 28 (3): 171–175. DOI: 10.1159/000238072 | wrong study design | no |
| 46 | Moss J., Price V., Paulus S. et al. Pharmacokinetics of a continuous infusion of piperacillin/tazobactam to children using an elastomeric pump (poppet study): Pilot data from double lumen central lines. Arch. Dis. Child. 2019;104:6 doi:10.1136/archdischild-2019-esdppp.107 | wrong publication type | no |
| 47 | Munckhof WJ, Carney J, Neilson G, et al. Continuous infusion of ticarcillin-clavulanate for home treatment of serious infections: clinical efficacy, safety, pharmacokinetics and pharmacodynamics. Int J Antimicrob Agents. 2005;25(6):514-522. doi:10.1016/j.ijantimicag.2005.02.008 | not the target population | no |
| 48 | Naik B. I., Roger C., Ikeda K. et al. Comparative total and unbound pharmacokinetics of cefazolin administered by bolus versus continuous infusion in patients undergoing major surgery: a randomized controlled trial. BJA: British Journal of Anaesthesia, 2017;118(6): 876–882. https://doi.org/10.1093/bja/aex026 | not the target population | no |
| 49 | Nichols KR, Knoderer CA, Cox EG, Kays MB. System-wide implementation of the use of an extended-infusion piperacillin/tazobactam dosing strategy: feasibility of utilization from a children's hospital perspective. Clin Ther. 2012;34(6):1459-1465. doi:10.1016/j.clinthera.2012.05.005 | wrong outcome | no |
| 50 | Oualha M., Prim B., Hirt D. et al. B-lactams in critically ill children with renal failure and continuous renal replacement therapy: Dosing and exposure. Ann. Intensive Care. 2020;10 Supplement 1 | the outcomes could not be pooled with others, no data about dosing | no |
| 51 | Paice, Kelli; Girdwood, Sonya Tang; Pavia, Kathryn et al. 38: Critically ill children with severe sepsis often have sub-target meropenem levels early in therapy. Critical Care Medicine 51(1):p 19, January 2023. \| DOI: 10.1097/01.ccm.0000906028.55585.5f | wrong publication type | no |
| 52 | Pea F, Viale P, Damiani D, et al. Ceftazidime in acute myeloid leukemia patients with febrile neutropenia: helpfulness of continuous intravenous infusion in maximizing pharmacodynamic exposure. Antimicrob Agents Chemother. 2005;49(8):3550-3553. doi:10.1128/AAC.49.8.3550-3553.2005 | not the target population | no |
| 53 | Pédeboscq S, Dubau B, Frappier S, et al. Comparaison de deux schémas d'administration (continu ou discontinu) d'un antibiotique temps-dépendant: la Tazocilline [Comparison of 2 administration protocols (continuous or discontinuous) of a time-dependent antibiotic, Tazocin]. Pathol Biol (Paris). 2001;49(7):540-547. doi:10.1016/s0369-8114(01)00210-3 | not the target population | no |
| 54 | Philpott, C. D.; Droege, C. A.; Droege, M. E. et al. Pharmacokinetics and Pharmacodynamics of Extended-Infusion Cefepime in Critically Ill Patients Receiving Continuous Renal Replacement Therapy: A Prospective, Open-Label Study. Pharmacotherapy. 2019;39(11): 1066-1076. doi: 10.1002/phar.2332 | not the target population | no |
| 55 | Philpott C., Droege C., Healy D. et al. Extended-infusion cefepime pharmacokinetics/pharmacodynamics in continuous renal replacement therapy.  Crit. Care Med. 2019 47:1 Supplement 1 | not the target population | no |
| 56 | Plasse J.-C., Chabloz C., Terrier A., Bellon G. To the editor: Is it safe to administer a continuous infusion of ceftazidime (Fortum®) prepared for 24 hours in cystic fibrosis (CF) patients? [1] Pediatr. Pulmonol. 2002 33:3 (232-233) | wrong study design | no |
| 57 | Prazak A.M., Alexander D. Evaluation of meropenem continuousinfusion clearance in burn patients Crit. Care Med. 2018 46 (474) Supplement 1 | wrong publication type | no |
| 58 | Prazak A.M., Alexander D., Cochran A. Evaluation of nafcillin continuous-infusion clearance in pediatric burn patients 2016 44:12 (305) Supplement 1 doi:10.1097/01.ccm.0000509591.32744.5a | wrong publication type | no |
| 59 | G. Ragonnet; O. Hanafia1; N. Vanel et al. Optimization of beta-lactam antibiotics exposure in pediatric intensive care unit: Protocolization of continuous infusion. Oral Communication Abstracts DOI: 10.1111/fcp.12905 | the outcomes could not be pooled with others (data based on antibiotic samples, not patients) | yes |
| 60 | Richter DC, Frey O, Röhr A, et al. Therapeutic drug monitoring-guided continuous infusion of piperacillin/tazobactam significantly improves pharmacokinetic target attainment in critically ill patients: a retrospective analysis of four years of clinical experience. Infection. 2019;47(6):1001-1011. doi:10.1007/s15010-019-01352-z | not the target population | no |
| 61 | Riethmueller J, Junge S, Schroeter TW et al. Continuous vs thrice-daily ceftazidime for elective intravenous antipseudomonal therapy in cystic fibrosis. Infection, 2009, 37(5), 418‐423. doi: 10.1007/s15010-009-8116-5 | not the target population | no |
| 62 | Roberts JA, Boots R, Rickard CM, et al. Is continuous infusion ceftriaxone better than once-a-day dosing in intensive care? A randomized controlled pilot study. J Antimicrob Chemother. 2007;59(2):285-291. doi:10.1093/jac/dkl478 | not the target population | no |
| 63 | Roberts JA, Roberts MS, Robertson TA, Dalley AJ, Lipman J. Piperacillin penetration into tissue of critically ill patients with sepsis--bolus versus continuous administration?. Crit Care Med. 2009;37(3):926-933. doi:10.1097/CCM.0b013e3181968e44 | not the target population | no |
| 64 | Roger C, Cotta MO, Muller L, et al. Impact of renal replacement modalities on the clearance of piperacillin-tazobactam administered via continuous infusion in critically ill patients. Int J Antimicrob Agents. 2017;50(2):227-231. doi:10.1016/j.ijantimicag.2017.03.018 | not the target population | no |
| 65 | Russell NJ, Stöhr W, Plakkal N, et al. Patterns of antibiotic use, pathogens, and prediction of mortality in hospitalized neonates and young infants with sepsis: A global neonatal sepsis observational cohort study (NeoOBS). PLoS Med. 2023;20(6):e1004179. Published 2023 Jun 8. doi:10.1371/journal.pmed.1004179 | wrong outcome | no |
| 66 | Sanz Codina M., Gatti M., Troisi C. et al. Relationship between Pharmacokinetic/Pharmacodynamic Target Attainment and Microbiological Outcome in Critically Ill COVID-19 Patients with Documented Gram-Negative Superinfections Treated with TDM-Guided Continuous-Infusion Meropenem.Pharmaceutics 2022 14:8 Article Number 1585. doi: 10.3390/pharmaceutics14081585 | not the target population | no |
| 67 | Schuster KM, Wilson D, Schulman CI, Pizano LR, Ward CG, Namias N. Continuous-infusion oxacillin for the treatment of burn wound cellulitis. Surg Infect (Larchmt). 2009;10(1):41-45. doi:10.1089/sur.2007.081 | not the target population | no |
| 68 | Tamma P.D., Jenh A.M., Milstone A.M. Prolonged β-lactam infusion for gram-negative infections. Pediatr. Infect. Dis. J. 2011;30:4(336-337) doi:10.1097/INF.0b013e31820ef3e5 | wrong publication type | no |
| 69 | Comparison of time above MIC as a PK/PD outcome of Extended vs Bolus Infusion of Piperacillin in Children, A Randomized Controlled Trial <https://trialsearch.who.int> Indentifier: TCTR20190904002 | wrong poblication type | no |
| 70 | Thalhammer F., Traunmuller F., El Menyawi I. Continuous infusion versus intermittent administration of meropenem in critically ill patients J. Antimicrob. Chemother. 1999; 43:4 (523-527) doi:10.1093/jac/43.4.523 | not the target population | no |
| 71 | Tichy M., Seidelman J., Lewis S.S., Drew R.H., Sarubbi C. Continuous vs. intermittent intraoperative infusion of cefazolin on surgical site infections (SSIS) and acute kidney injury in patients undergoing cardiac procedures. Open Forum Infect. Dis. 2019;6 (S447-S448) Supplement 2 | not the target population | no |
| 72 | Tschumper E., Dupuis K., McCrory K., Pitts W. Evaluation of prolonged versus continuous infusions of piperacillin/tazobactam in the setting of drug shortages.  JACCP J.Am. Coll. Clin. Pharm. 2021 4:9 (1226-1227) doi: 10.1002/jac5.1481 | not the target population | no |
| 73 | Turner R., Biondo L.R., Slain D., Phillips U., Cardenas S.C., Moffett K. A randomized pilot study of continuous versus intermittent infusion piperacillintazobactam for the treatment of pulmonary exacerbations in patients with cystic fibrosis. Pediatr. Pulmonol. 2013;48 (326) SUPPL. 36 | wrong publication type | no |
| 74 | van Boekholt A., Fleuren H., Mouton J. et al. Serum concentrations of amoxicillin in neonates during continuous intravenous infusion. Eur. J. Clin. Microbiol. Infect. Dis. 2016 35:6 (1007-1012) | no comparator | yes |
| 75 | Vinks, A. A., Brimicombe, R. W., Heijerman, H. G., Bakker, W. Continuous infusion of ceftazidime in cystic fibrosis patients during home treatment: clinical outcome, microbiology and pharmacokinetics. J Antimicrob Chemother. 1997 Jul;40(1):125-33. doi: 10.1093/jac/40.1.125. | not the target population | no |
| 76 | Visser L.G., Arnouts P., Van Furth R., Mattie H., Van den Broek P.J. Clinical pharmacokinetics of continuous intravenous administration of penicillins. Clin. Infect. Dis. 1993 17:3 (491-495). | not the target population | no |
| 77 | Wang Z, Bi J, You D, et al. Improving the efficacy for meropenem therapy requires a high probability of target attainment in critically ill infants and children. Front Pharmacol. 2022;13:961863. Published 2022 Oct 5. doi:10.3389/fphar.2022.961863 | no comparator | no |
| 78 | Zeller V, Durand F, Kitzis MD, et al. Continuous cefazolin infusion to treat bone and joint infections: clinical efficacy, feasibility, safety, and serum and bone concentrations. Antimicrob Agents Chemother. 2009;53(3):883-887. doi:10.1128/AAC.00389-08 | not the target population | no |
| 79 | Zhou P, Cheng Y, Cao G, et al. The OBTAINS study: A nationwide cross-sectional survey on the implementation of extended or continuous infusion of β-lactams and vancomycin among neonatal sepsis patients in China [published correction appears in Front Pharmacol. 2022 Dec 16;13:1120032]. Front Pharmacol. 2022;13:1001924. Published 2022 Oct 10. doi:10.3389/fphar.2022.1001924 | wrong outcome | no |
| 80 | Zobell JT, Young DC, Chatfield BA. Intermittent and extended-infusion beta-lactam utilization in cystic fibrosis. Pediatr Pulmonol. 2013;48(6):622-623. doi:10.1002/ppul.22641 | wrong publication type | no |

**Results**

**The hypotheses of the RCTs for calculating the number of patients**

Chongcharoenyanon et al.^13^: The hypothesis was based on the proportion of participants in the extended and intermittent bolus group who had Cmid above MIC were 82% and 47%, respectively.

Fuentes et al. (NCT03019965)^14^: no information

Pelkonen et al.^15^: They hypothesised that the addition of paracetamol therapy and the slow infusion can improve the prognosis (death and prevention of neurological sequelae) of bacterial meningitis by 15% compared to the bolus group.

Savonius et al.^16^: They hypothesised that there would be a 13% decrease in mortality in the extended infusion group.

Shabaan et al.^17^: sample size calculation was not performed because of the paucity of similar previous studies.

Solórzano-Santoz et al.^18^: The objective of the study was to evaluate the clinical efficacy of the continuous infusion compared to the intermittent infusion. For sample size calculation 15% difference was assumed between the two groups.

Wang et al.^19^: no information

a


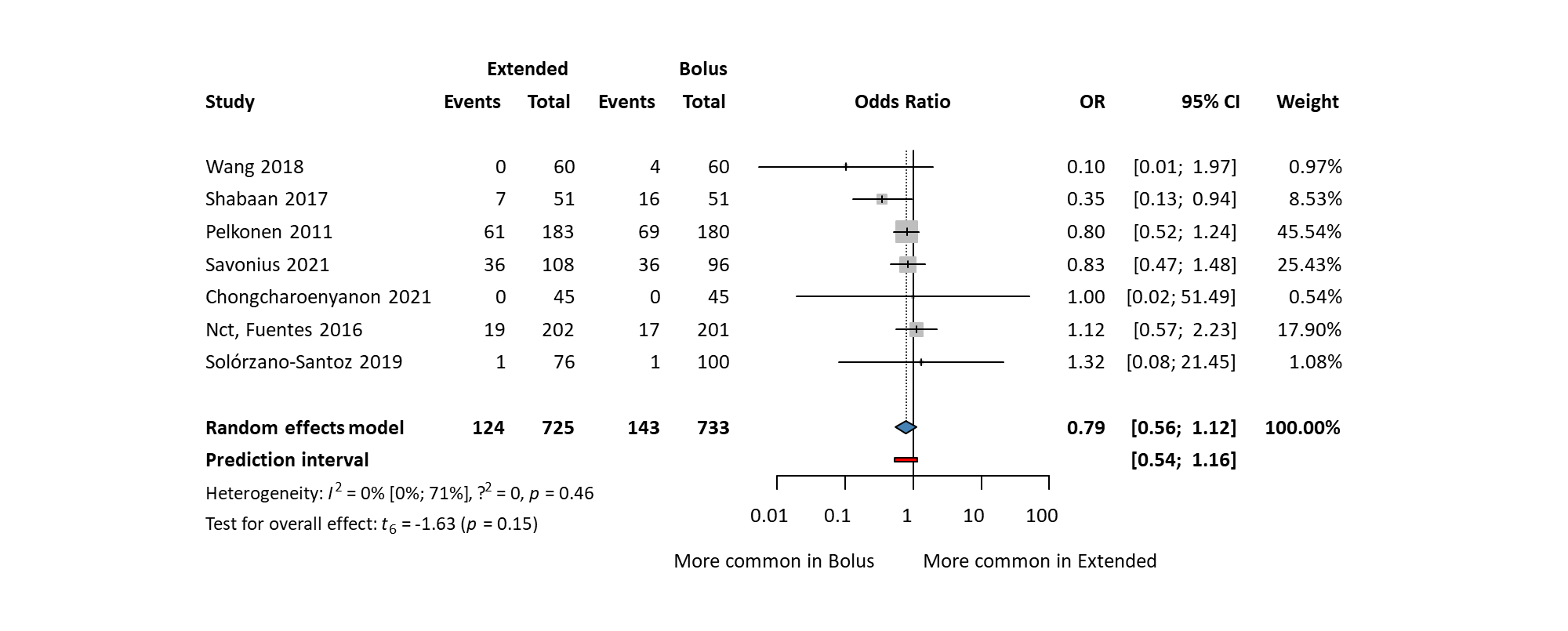


b


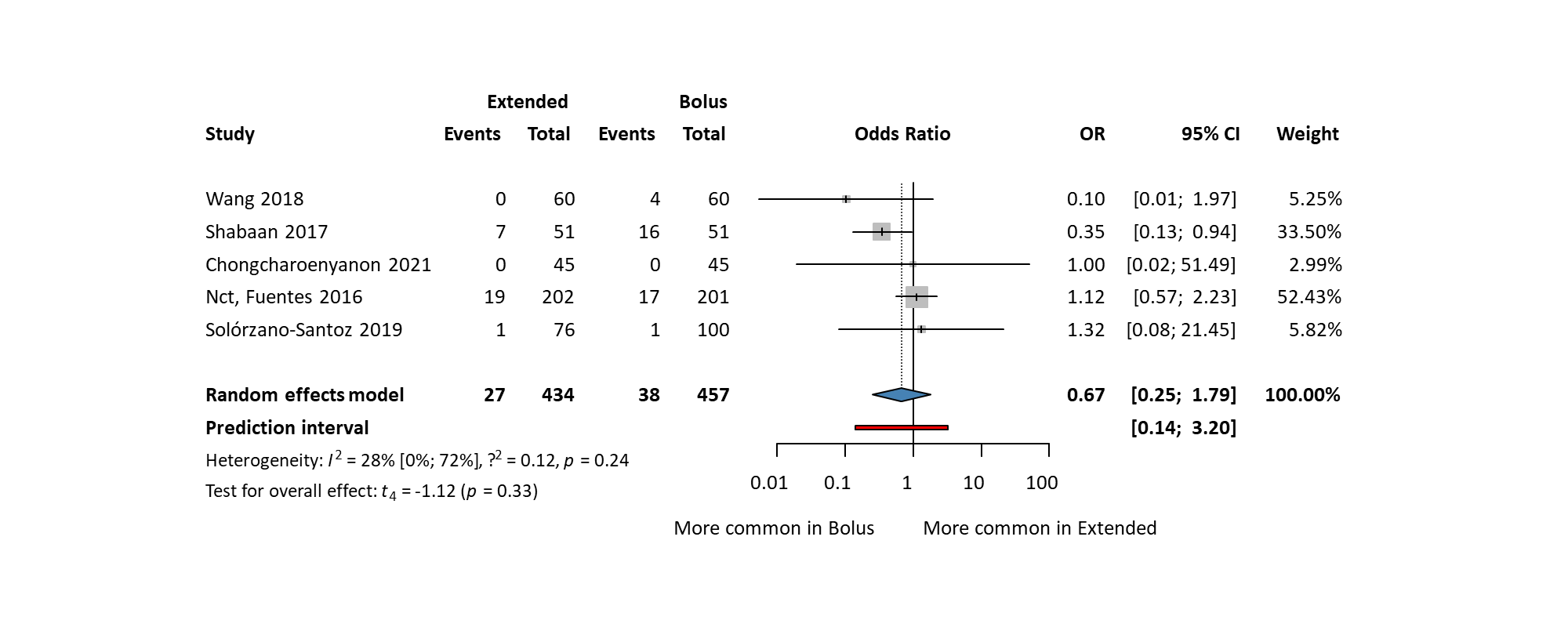


**Figure 1S.** Forest plots of all-cause mortality among paediatric patients treated with extended versus bolus infusion of beta-lactams, including RCTs. (a) all studies (b) without meningitis studies
CI confidence interval; OR odds ratio

In the study of Pelkonen et al.^15^ patients were randomly allocated into four groups (cefotaxime extended infusion plus oral paracetamol, cefotaxime extended infusion plus oral placebo, cefotaxime bolus infusion plus oral paracetamol, cefotaxime bolus infusion plus oral placebo), and we used the data of the two groups treated with paracetamol. In addition, in the extended group the antibiotic was administered only in the first 24 hours continuously, and after 24 hours all the patients received the bolus regimen. In the study of Savonius et al.^16^ patients were randomly allocated into two groups (cefotaxime extended infusion plus oral paracetamol, cefotaxime bolus infusion plus oral placebo), and in the extended group cefotaxime was given in the first 96 hours continuously, then everybody received the bolus regimen.


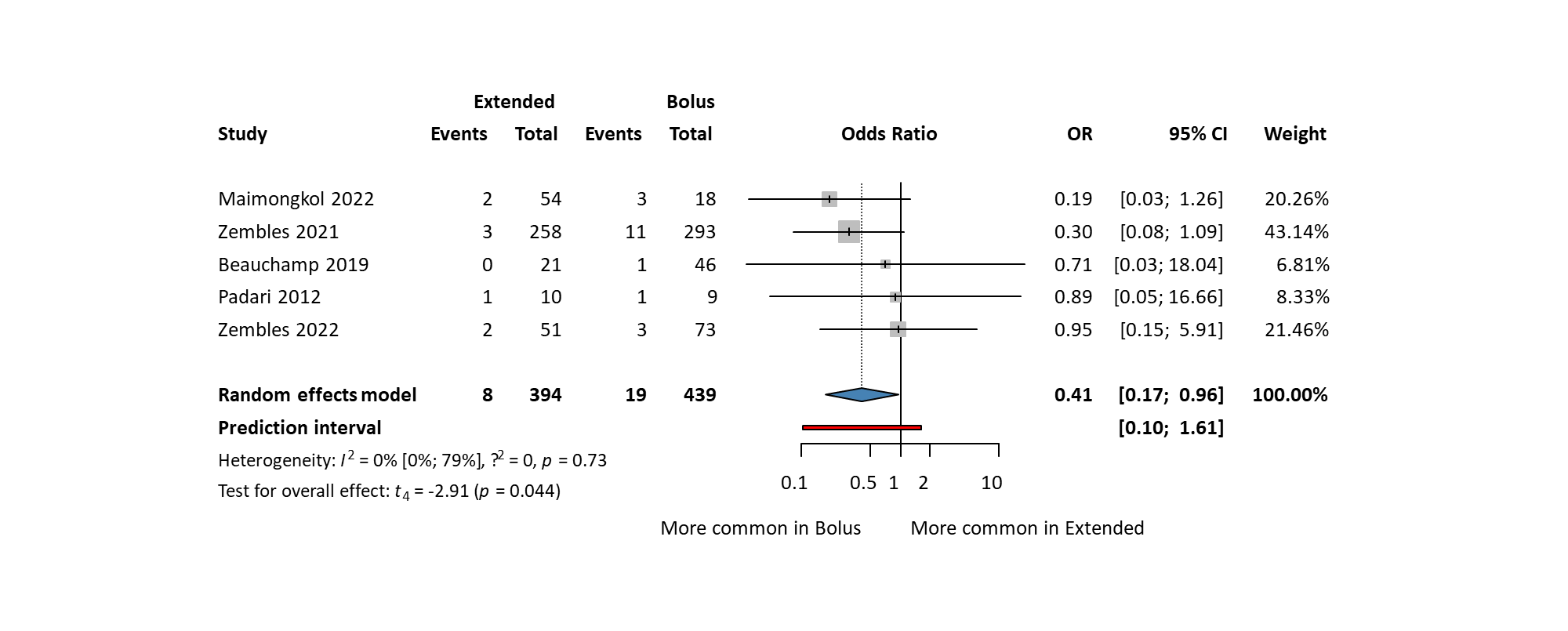


**Figure 2S.** Forest plot of all-cause mortality among paediatric patients treated with extended versus bolus infusion of beta-lactams, including non-RCTs. CI confidence interval; OR odds ratio


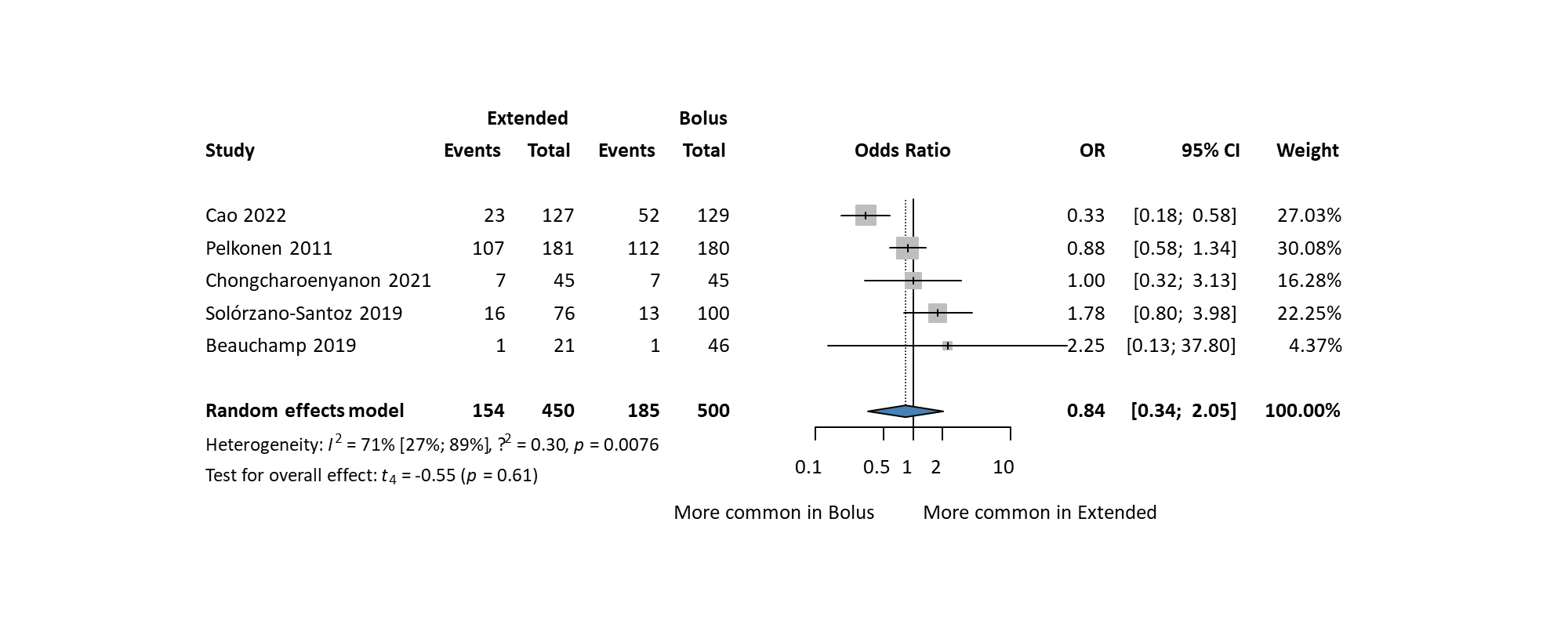


**Figure 3S.** Forest plot of treatment failure on the 3rd day among paediatric patients treated with extended versus bolus infusion of beta-lactams. CI confidence interval; OR odds ratio

The definition of treatment failure was heterogeneous: the absence of defervescence and/or white blood cell normalization and/or culture clearance, the lack of normal blood pressure and/or heart rate and/or stable hemodynamics, the presence of dyspnea, the insufficient intestinal tolerance, and need for invasive mechanical ventilation on the third day. Beauchamp et al.^20^ included the absence of culture clearance in the definition of unsuccessful treatment, while the others only refered to the presence of clinical symptoms.


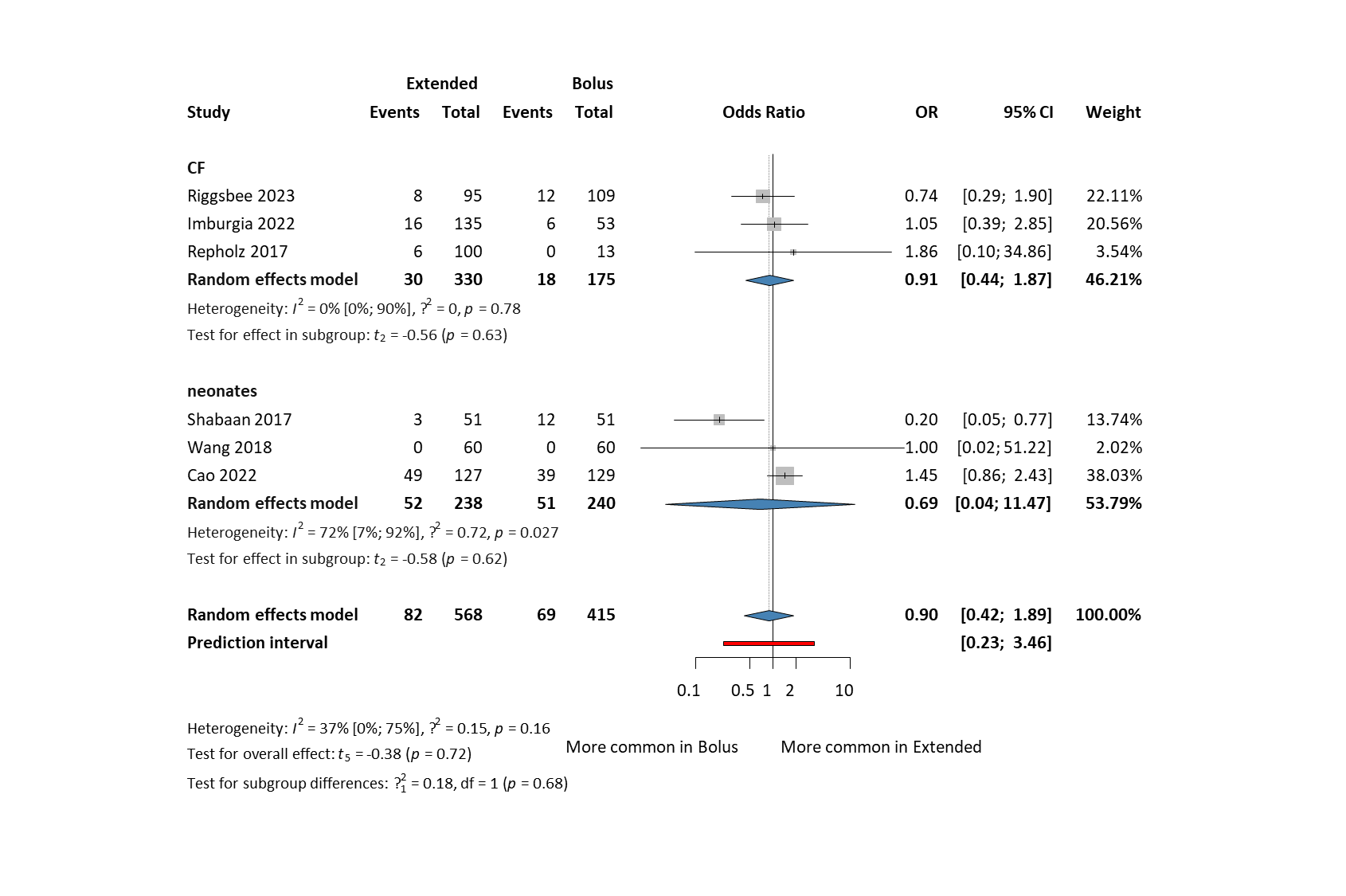


**Figure 4S.** Forest plots of acute kidney injury (AKI), subgroup analysis of cystic fibrosis (CF) patients and neonates. CI confidence interval; OR odds ratio


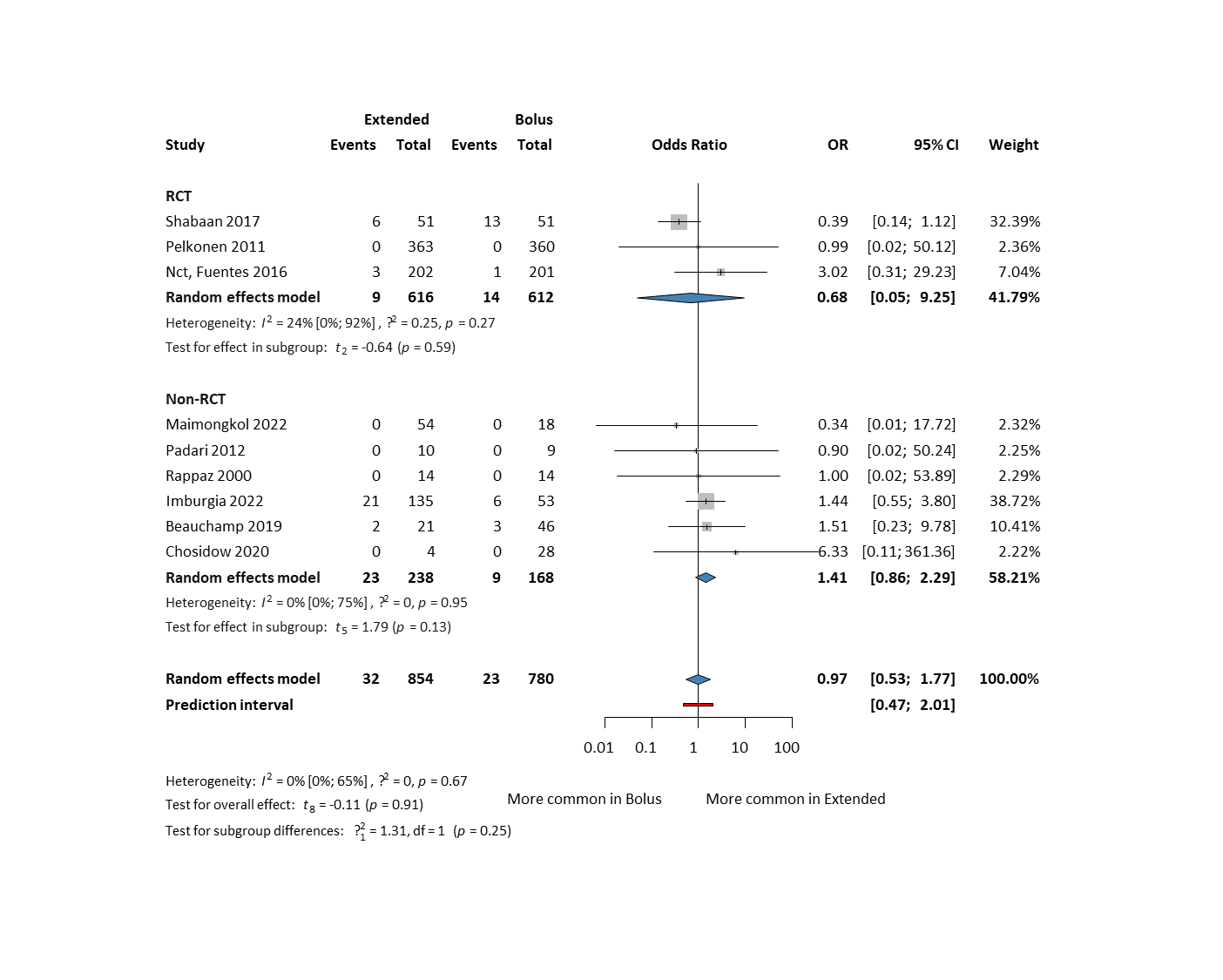


**Figure 5S.** Forest plots of all adverse events among paediatric patients treated with extended versus bolus infusion of beta-lactams, subgroup analysis of RCTs and non-RCTs. CI confidence interval; OR odds ratio

The identification of all adverse events could be problematic and variable in the studies. In the articles gastrointestinal side effects (diarrhea, nausea, vomiting), rash, hypersensitivity, shacking/chills, haematologic toxicity, neurologic toxicity (encephalopathy, seizure, EEG changes), drug-related laboratory abnormalities or any clinical or biological toxicity were collected.

a


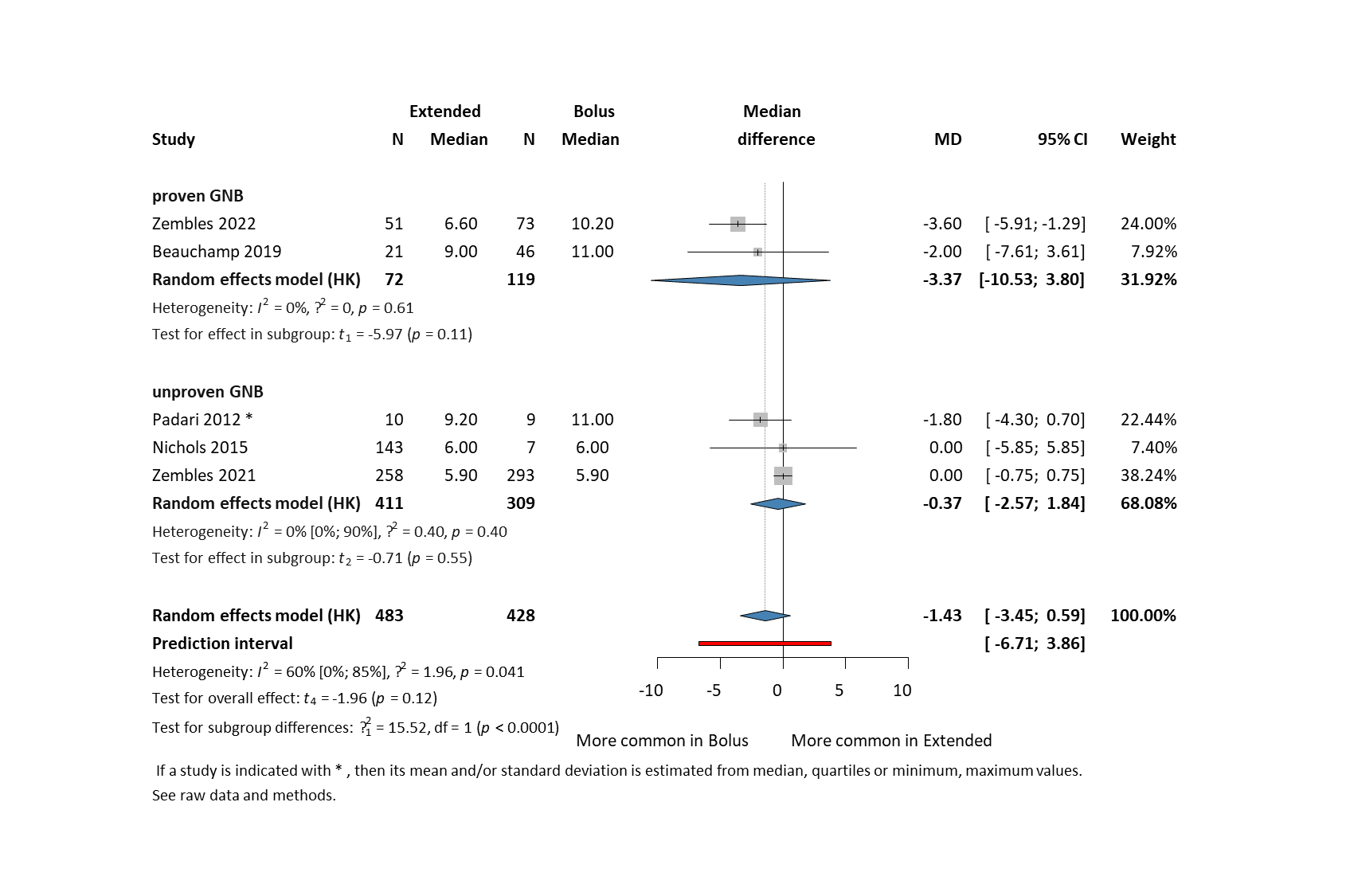


b


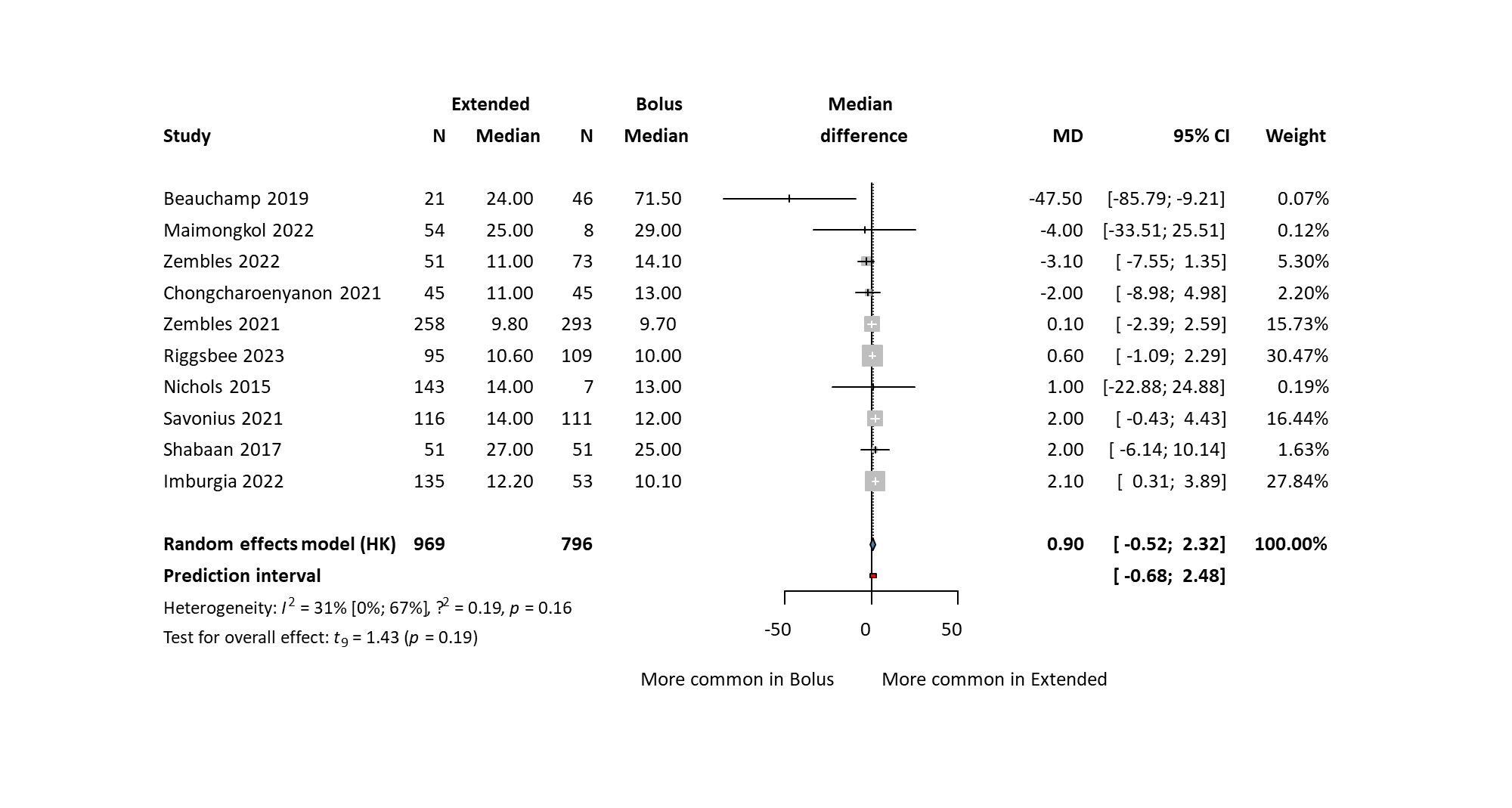


c


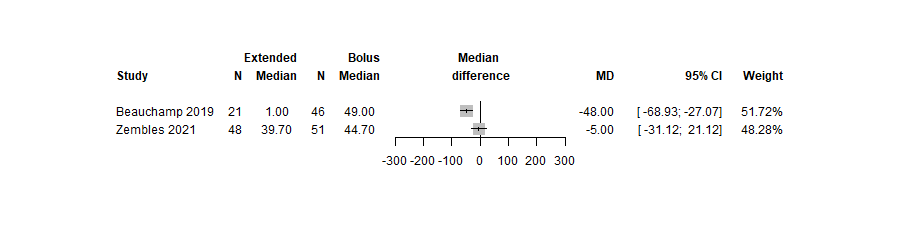


**Figure 6S.** Forest plots of (a) the duration of the antibiotic (b) LOS (c) PICU length of stay among paediatric patients treated with extended versus bolus infusion of beta-lactams. CI confidence interval; GNB Gram-negative bacteremia; LOS length of stay; OR odds ratio; PICU paediatric intensive care unit

**Table 3S. Raw data table for the duration of antibiotic course**

| Study | N (EI) | Mean (EI) | SD (EI) | Median (EI) | Q1 (EI) | Q3 (EI) | Min (EI) | Max (EI) | N (SI) | Mean (SI) | SD (SI) | Median (SI) | Q1 (SI) | Q3 (SI) | Min (SI) | Max (SI) |
| --- | --- | --- | --- | --- | --- | --- | --- | --- | --- | --- | --- | --- | --- | --- | --- | --- |
| Beauchamp 2019 | 21 |  |  | 9.0 | 3.0 | 15.0 |  |  | 46 |  |  | 11.0 | 4.0 | 15.0 |  |  |
| Nichols 2015 | 143 |  |  | 6.0 | 3.0 | 10.0 | 1 | 35 | 7 |  |  | 6.0 | 3.0 | 13.0 | 2 | 15 |
| Padari 2012 * | 10 | 9.2 | 3.5 |  |  |  |  |  | 9 | 11 | 1.9 |  |  |  |  |  |
| Zembles 2021 | 258 |  |  | 5.9 | 4.2 | 9.2 |  |  | 293 |  |  | 5.9 | 4.0 | 8.8 |  |  |
| Zembles 2022 | 51 |  |  | 6.6 | 4.3 | 9.9 |  |  | 73 |  |  | 10.2 | 5.6 | 14.3 |  |  |

EI extended infusion; Max maximum value; Min minimum value; N number; SD standard deviation; SI short-term intermittent infusion; Q1 first quartile; Q3 third quartile

**Table 4S. Raw data table for the length of hospital stay**

| Study | N (EI) | Median (EI) | Q1 (EI) | Q3 (EI) | Min (EI) | Max (EI) | N (SI) | Median (SI) | Q1 (SI) | Q3 (SI) | Min (SI) | Max (SI) |
| --- | --- | --- | --- | --- | --- | --- | --- | --- | --- | --- | --- | --- |
| Beauchamp 2019 | 21 | 24.0 | 10.0 | 46.5 |  |  | 46 | 71.5 | 8.0 | 156.8 |  |  |
| Chongcharoenyanon 2021 | 45 | 11.0 | 6.0 | 27.0 |  |  | 45 | 13.0 | 7.0 | 25.0 |  |  |
| Imburgia 2022 | 135 | 12.2 | 9.3 | 14.7 |  |  | 53 | 10.1 | 7.3 | 13.6 |  |  |
| Maimongkol 2022 | 54 | 25.0 | 12.0 | 46.0 |  |  | 8 | 29.0 | 10.0 | 56.0 |  |  |
| Nichols 2015 | 143 | 14.0 | 7.0 | 31.0 | 2 | 160 | 7 | 13.0 | 4.0 | 55.0 | 2 | 55 |
| Riggsbee 2023 | 95 | 10.6 | 7.0 | 14.1 |  |  | 109 | 10.0 | 7.0 | 13.0 |  |  |
| Savonius 2021 | 116 | 14.0 | 10.0 | 20.0 |  |  | 111 | 12.0 | 10.0 | 20.0 |  |  |
| Shabaan 2017 | 51 | 27.0 | 15.0 | 39.0 |  |  | 51 | 25.0 | 15.0 | 36.0 |  |  |
| Zembles 2021 | 258 | 9.8 | 5.7 | 21.3 |  |  | 293 | 9.7 | 5.7 | 24.2 |  |  |
| Zembles 2022 | 51 | 11.0 | 6.3 | 19.3 |  |  | 73 | 14.1 | 8.2 | 23.7 |  |  |

EI extended infusion; Max maximum value; Min minimum value; N number; SI short-term intermittent infusion; Q1 first quartile; Q3 third quartile

The study of Beauchamp et al.^20^ demonstrated a spurious effect in LOS and PICU length of stay plots, because of the complicated nature and comorbidities of the study patients. Length of stay may not be clearly related to infection or antibiotic infusion strategy. Regarding the duration of the antibiotic, if a study is indicated with *, then its mean and/or standard deviaton is estimated from median, quartiles or minimum, maximum values. We did not take in consideration the CF patients because of the predefined protocols in their care. There was no difference between the EI and SI group examining LOS and the duration of the antibiotic course.

a

b

**Figure 7S.** Funnel plots of studies with all-cause mortality outcome. (a) all studies (b) without meningitis studies

a

b

**Figure 8S.** Influential analyses of studies with all-cause mortality outcome. (a) all studies (b) without meningitis studies. Explanations of the plots are in ‘Detailed description of synthesis methods’.

a

b

**Figure 9S.** Leave-one-out analyses, sorted by effect size plots of studies with all-cause mortality outcome. (a) all studies (b) without meningitis studies. Explanations of the plots are in ‘Detailed description of synthesis methods’.


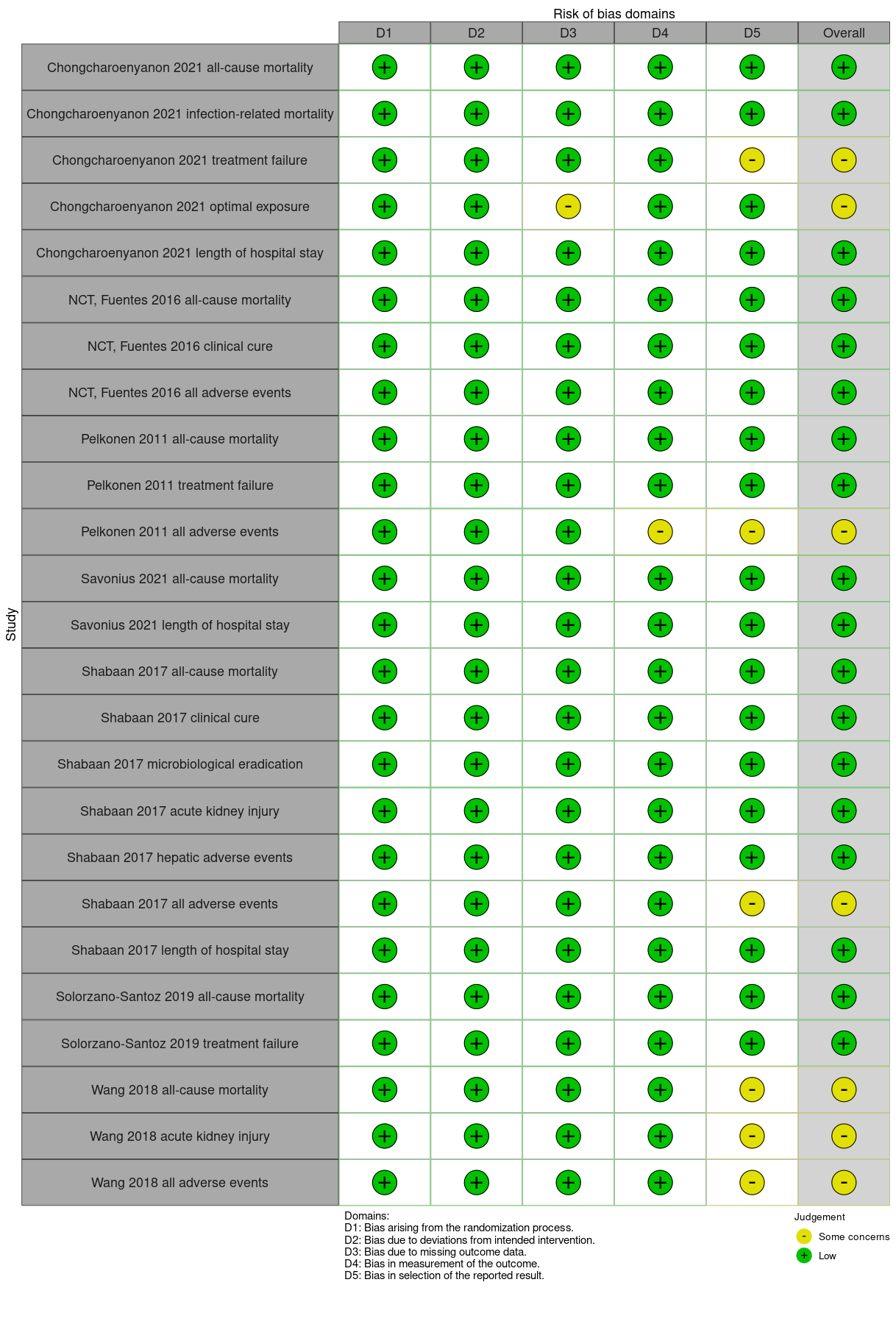
**Figure 10S.** Risk of bias assessment of each outcome using the revised Cochrane risk-of-bias tool for RCTs (RoB2)


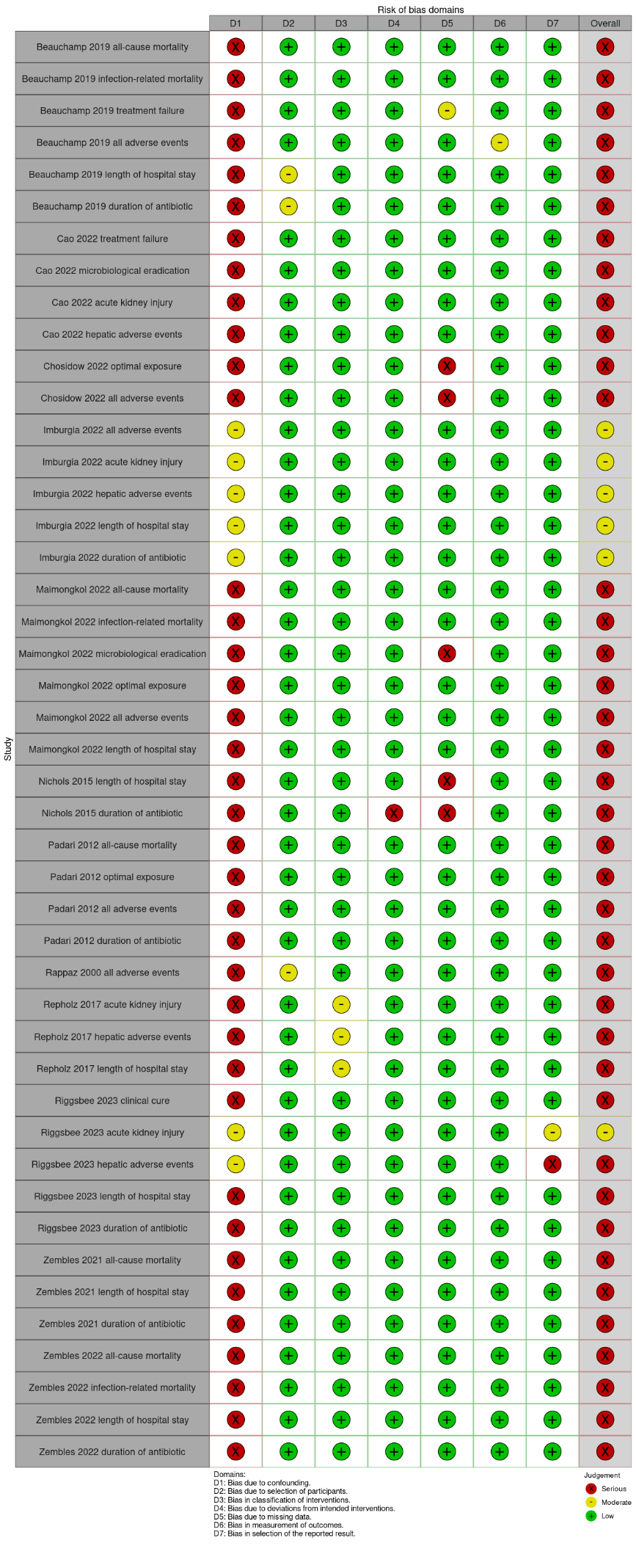


**Figure 11S.** Risk of bias assessment of each outcome using the revised Cochrane risk-of-bias tool non-RCTs (ROBINS-I)

**Table 5S. Summary of findings: Grading of Recommendations, Assessment, Development and Evaluations (GRADE) framework.**

**CF: cystic fibrosis; CI: confidence interval; fT>MIC percent of time free drug remains above the minimum inhibitory concentration; MD: mean difference; MIC minimal inhibitory concentration; No number; OR: odds ratio; PICU: Paediatric Intensive Care Unit; RCT randomised controlled trial**

| **Certainty assessment** | | | | | | | **№ of patients** | | **Effect** | | **Certainty** | **Importance** |
| --- | --- | --- | --- | --- | --- | --- | --- | --- | --- | --- | --- | --- |
| **№ of studies** | **Study design** | **Risk of bias** | **Inconsistency** | **Indirectness** | **Imprecision** | **Other considerations** | **prolonged infusion** | **intermittent, short-term infusion** | **Relative (95% CI)** | **Absolute (95% CI)** |  |  |
| **50-100% fT>MIC, measured MIC or using MIC cutoff ≤2 mg/L for susceptible bacteria 1** | | | | | | | | | | | | |
| 3 | observational studies | very serious^a^ | serious^b^ | not serious | very serious^c^ | dose response gradient | 70/75 (93.3%) | 28/37 (75.7%) | **OR 2.99** (0.28 to 31.81) | **146 more per 1 000** (from 291 fewer to 233 more) | ⨁◯◯◯ Very low | IMPORTANT |
| **50-100% fT>MIC, measured MIC or using MIC cutoff ≤2 mg/L for susceptible bacteria 2** | | | | | | | | | | | | |
| 3 | observational studies | very serious^a^ | serious^b^ | not serious | very serious^c^ | dose response gradient | 44/72 (61.1%) | 20/37 (54.1%) | **OR 1.97** (0.32 to 12.15) | **158 more per 1 000** (from 267 fewer to 394 more) | ⨁◯◯◯ Very low | IMPORTANT |

**Explanations**

a. High risk of bias because of the confounding factors and missing data

b. Clinically heterogeneous population

c. Very small sample size, wide CI

| **Certainty assessment** | | | | | | | **№ of patients** | | **Effect** | | **Certainty** | **Importance** |
| --- | --- | --- | --- | --- | --- | --- | --- | --- | --- | --- | --- | --- |
| **№ of studies** | **Study design** | **Risk of bias** | **Inconsistency** | **Indirectness** | **Imprecision** | **Other considerations** | **prolonged infusion** | **intermittent, short-term infusion** | **Relative (95% CI)** | **Absolute (95% CI)** |  |  |
| **All-cause mortality RCT OR** | | | | | | | | | | | | |
| 6 | randomised trials | not serious | serious^a^ | not serious | not serious | none | 124/725 (17.1%) | 143/733 (19.5%) | **OR 0.79** (0.56 to 1.12) | **34 fewer per 1 000** (from 76 fewer to 18 more) | ⨁⨁⨁◯ Moderate | CRITICAL |
| **All-cause mortality RCT without meningitis OR** | | | | | | | | | | | | |
| 4 | randomised trials | not serious | serious^a^ | not serious | serious^b^ | none | 27/434 (6.2%) | 38/457 (8.3%) | **OR 0.67** (0.25 to 1.79) | **26 fewer per 1 000** (from 61 fewer to 57 more) | ⨁⨁◯◯ Low | CRITICAL |
| **All-cause mortality non-RCT OR** | | | | | | | | | | | | |
| 6 | observational studies | very serious^c^ | serious^a^ | not serious | serious^b^ | none | 8/394 (2.0%) | 19/439 (4.3%) | **OR 0.41** (0.17 to 0.96) | **25 fewer per 1 000** (from 36 fewer to 2 fewer) | ⨁◯◯◯ Very low | CRITICAL |
| **Infection-related mortality (without meningitis) OR** | | | | | | | | | | | | |
| 4 | observational studies | very serious^c^ | not serious | not serious | very serious^d^ | none | 1/171 (0.6%) | 3/182 (1.6%) | **OR 0.52** (0.24 to 1.12) | **8 fewer per 1 000** (from 12 fewer to 2 more) | ⨁◯◯◯ Very low | CRITICAL |
| **All-cause mortality, meropenem treated patients** | | | | | | | | | | | | |
| 4 | observational studies | serious^c^ | not serious | not serious | very serious^d^ | none | 10/175 (5.7%) | 24/138 (17.4%) | **OR 0.31** (0.13 to 0.73) | **113 fewer per 1 000** (from 147 fewer to 41 fewer) | ⨁◯◯◯ Very low | CRITICAL |
| **All-cause mortality, neonates** | | | | | | | | | | | | |
| 3 | observational studies | serious^c^ | not serious | not serious | very serious^d^ | none | 8/121 (6.6%) | 21/120 (17.5%) | **OR 0.34** (0.08 to 1.40) | **108 fewer per 1 000** (from 158 fewer to 54 more) | ⨁◯◯◯ Very low | CRITICAL |

#### Explanations

a. Clinically heterogeneous population

b. Small sample size, wide CI

c. Non-RCTs: high risk of bias because of the confounding factors

d. Very small sample size

| **Certainty assessment** | | | | | | | **№ of patients** | | **Effect** | | **Certainty** | **Importance** |
| --- | --- | --- | --- | --- | --- | --- | --- | --- | --- | --- | --- | --- |
| **№ of studies** | **Study design** | **Risk of bias** | **Inconsistency** | **Indirectness** | **Imprecision** | **Other considerations** | **prolonged infusion** | **intermittent, short-term infusion** | **Relative (95% CI)** | **Absolute (95% CI)** |  |  |
| **Clinical cure OR** | | | | | | | | | | | | |
| 3 | observational studies | serious^a^ | very serious^b^ | serious^c^ | very serious^d^ | none | 233/320 (72.8%) | 232/325 (71.4%) | **OR 1.20** (0.17 to 8.71) | **36 more per 1 000** (from 416 fewer to 242 more) | ⨁◯◯◯ Very low | IMPORTANT |
| **Microbiological eradication OR** | | | | | | | | | | | | |
| 3 | observational studies | very serious^e^ | not serious | not serious | not serious | none | 169/191 (88.5%) | 140/183 (76.5%) | **OR 3.18** (2.24 to 4.51) | **147 more per 1 000** (from 114 more to 171 more) | ⨁⨁◯◯ Low | CRITICAL |
| **Treatment failure OR** | | | | | | | | | | | | |
| 5 | observational studies | serious^e^ | serious^b^ | serious^f^ | serious^g^ | none | 154/450 (34.2%) | 185/500 (37.0%) | **OR 0.84** (0.34 to 2.05) | **40 fewer per 1 000** (from 204 fewer to 176 more) | ⨁◯◯◯ Very low | IMPORTANT |

#### Explanations

a. Non-RCTs: high risk of bias because of the confounding factors

b. Clinically heterogenous population

c. In Riggsbee et. al. study: it was an additional outcome.

d. Very wide CI and small number of patients

e. Non-RCTs: high risk of bias because of the confounding factors, missing data

f. In Chongcharoenyanon et al. study: it was an additional outcome.

g. Wide CI

| **Certainty assessment** | | | | | | | **№ of patients** | | **Effect** | | **Certainty** | **Importance** |
| --- | --- | --- | --- | --- | --- | --- | --- | --- | --- | --- | --- | --- |
| **№ of studies** | **Study design** | **Risk of bias** | **Inconsistency** | **Indirectness** | **Imprecision** | **Other considerations** | **prolonged infusion** | **intermittent, short-term infusion** | **Relative (95% CI)** | **Absolute (95% CI)** |  |  |
| **All adverse events, subgroups OR** | | | | | | | | | | | | |
| 6 | observational studies | serious^a^ | serious^b^ | not serious^c^ | not serious | all plausible residual confounding would suggest spurious effect, while no effect was observed^d^ | 23/238 (9.7%) | 9/168 (5.4%) | **OR 1.41** (0.86 to 2.29) | **20 more per 1 000** (from 7 fewer to 61 more) | ⨁⨁⨁◯ Moderate | IMPORTANT |
| **Acute kidney injury OR** | | | | | | | | | | | | |
| 5 | observational studies | serious^e^ | serious^f^ | not serious | serious^g^ | all plausible residual confounding would suggest spurious effect, while no effect was observed^d^ | 82/568 (14.4%) | 69/415 (16.6%) | **OR 0.90** (0.42 to 1.89) | **14 fewer per 1 000** (from 89 fewer to 107 more) | ⨁⨁◯◯ Low | IMPORTANT |
| **Hepatic adverse events OR** | | | | | | | | | | | | |
| 5 | observational studies | serious^e^ | serious^f^ | not serious | not serious | all plausible residual confounding would suggest spurious effect, while no effect was observed^d^ | 75/508 (14.8%) | 50/355 (14.1%) | **OR 1.11** (0.77 to 1.58) | **13 more per 1 000** (from 29 fewer to 65 more) | ⨁⨁⨁◯ Moderate | IMPORTANT |
| **Acute kidney injury subgroups (CF patients and neonates) OR** | | | | | | | | | | | | |
| 6 | observational studies | serious^e^ | not serious | not serious | serious^g^ | all plausible residual confounding would suggest spurious effect, while no effect was observed^d^ | 82/568 (14.4%) | 69/415 (16.6%) | **OR 0.90** (0.42 to 1.89) | **14 fewer per 1 000** (from 89 fewer to 107 more) | ⨁⨁⨁◯ Moderate | IMPORTANT |

**Explanations**

a. RoB: high

b. heterogenous population: patients with Gram-negatív bacteremia, PICU, neonates, CF patients

c. In some cases it was not a direct outcome

d. Non-measured effect

e. Non-RCTs: high risk of bias due to the confounding factors

f. Clinically heterogenous population

g. Wide CI, small number of patients

| **Certainty assessment** | | | | | | | **№ of patients** | | **Effect** | | **Certainty** | **Importance** |
| --- | --- | --- | --- | --- | --- | --- | --- | --- | --- | --- | --- | --- |
| **№ of studies** | **Study design** | **Risk of bias** | **Inconsistency** | **Indirectness** | **Imprecision** | **Other considerations** | **prolonged infusion** | **intermittent, short-term infusion** | **Relative (95% CI)** | **Absolute (95% CI)** |  |  |
| **Length of hospital stay** | | | | | | | | | | | | |
| 10 | observational studies | serious^a^ | serious^b^ | not serious | not serious | all plausible residual confounding would suggest spurious effect, while no effect was observed^c^ | 969 | 796 | - | MD **0.9 day more** (0.52 fewer to 2.32 more) | ⨁⨁⨁◯ Moderate | NOT IMPORTANT |
| **Duration of antibiotic (without CF patients)** | | | | | | | | | | | | |
| 5 | observational studies | serious^a^ | serious^b^ | serious^e^ | not serious | none | 483 | 428 | - | MD **1.43 day fewer** (3.45 fewer to 0.59 more) | ⨁◯◯◯ Very low | NOT IMPORTANT |

#### Explanations

a. Non-RCTs: high risk of bias because of the confounding factors, selection of participants and classification of interventions

b. Clinically heterogenous population

c. Beauchamp et al.: Length of stay may not be clearly related to infection or antibiotic infusion strategy.

e. The outcome was not the aim of the studies.

**REFERENCES**

1 Statistical Aspects of the Analysis of Data From Retrospective Studies of Disease. *JNCI: Journal of the National Cancer Institute* 1959; published online April. DOI:10.1093/jnci/22.4.719.

2 ROBINS J, GREENLAND S, BRESLOW NE. A GENERAL ESTIMATOR FOR THE VARIANCE OF THE MANTEL HAENSZEL ODDS RATIO. *Am J Epidemiol* 1986; **124**: 719–23.

3 Cooper H, Hedges L V, Valentine JC, editors. The handbook of research synthesis and meta-analysis, 2nd ed. New York,  NY,  US: Russell Sage Foundation, 2009.

4 J. Sweeting M, J. Sutton A, C. Lambert P. What to add to nothing? Use and avoidance of continuity corrections in meta-analysis of sparse data. *Stat Med* 2004; **23**: 1351–75.

5 McGrath S, Sohn H, Steele R, Benedetti A. Meta‐analysis of the difference of medians. *Biometrical Journal* 2020; **62**: 69–98.

6 Padari H, Metsvaht T, Kõrgvee LT, *et al.* Short versus long infusion of meropenem in very-low-birth-weight neonates. *Antimicrob Agents Chemother* 2012; **56**: 4760–4.

7 Imburgia TA, Engdahl SR, Pettit RS. Evaluation of the safety of cefepime prolonged infusions in pediatric patients with cystic fibrosis. *Pediatr Pulmonol* 2022; **57**: 919–25.

8 Knapp G, Hartung J. Improved tests for a random effects meta-regression with a single covariate. *Stat Med* 2003; **22**: 2693–710.

9 IntHout J, Ioannidis JP, Borm GF. The Hartung-Knapp-Sidik-Jonkman method for random effects meta-analysis is straightforward and considerably outperforms the standard DerSimonian-Laird method. *BMC Med Res Methodol* 2014; **14**: 25.

10 Paule RC, Mandel J. Consensus Values and Weighting Factors. *J Res Natl Bur Stand (1934)* 1982; **87**: 377.

11 Veroniki AA, Jackson D, Viechtbauer W, *et al.* Methods to estimate the between‐study variance and its uncertainty in meta‐analysis. *Res Synth Methods* 2016; **7**: 55–79.

12 Harrer M, Cuijpers P, Furukawa TA, Ebert DD. Doing Meta-Analysis with R, 1st edn. Boca Raton: Chapman and Hall/CRC, 2021 DOI:10.1201/9781003107347.

13 Chongcharoenyanon T, Wacharachaisurapol N, Anugulruengkitt S, *et al.* Comparison of piperacillin plasma concentrations in a prospective randomised trial of extended infusion versus intermittent bolus of piperacillin/tazobactam in paediatric patients. *International Journal of Infectious Diseases* 2021; **108**: 102–8.

14 Fuentes YDC. Efficacy of Betalactam Antibiotics in Prolonged Infusion Compared to Intermittent in Pediatric Patients With Sepsis. ClinicalTrials.gov Identifier: NCT03019965. https://beta.clinicaltrials.gov/study/NCT03019965 (accessed Aug 30, 2023).

15 Pelkonen T, Roine I, Cruzeiro ML, Pitkäranta A, Kataja M, Peltola H. Slow initial β-lactam infusion and oral paracetamol to treat childhood bacterial meningitis: a randomised, controlled trial. *Articles Lancet Infect Dis* 2011; **11**: 613–34.

16 Savonius O, Rugemalira E, Roine I, Cruzeiro ML, Peltola H, Pelkonen T. Extended Continuous β-Lactam Infusion with Oral Acetaminophen in Childhood Bacterial Meningitis: A Randomized, Double-blind Clinical Trial. *Clinical Infectious Diseases* 2021; **72**: 1738–44.

17 Shabaan AE, Nour I, Elsayed Eldegla H, Nasef N, Shouman B, Abdel-Hady H. Conventional Versus Prolonged Infusion of Meropenem in Neonates With Gram-negative Late-onset Sepsis: A Randomized Controlled Trial. *Pediatric Infectious Disease Journal* 2017; **36**: 358–63.

18 Solórzano-Santos F, Quezada-Herrera A, Fuentes-Pacheco Y, *et al.* Piperacillin/tazobactam in continuous infusion versus intermittent infusion in children with febrile neutropenia. *Revista de Investigacion Clinica* 2019; **71**: 283–90.

19 Li W, Shen-wang N, Ke-ran Z, Yang W, Li-li W, Fang D. Efficacy of prolonged intravenous infusion duration of meropenem on neonatal late-onset sepsis. *Guangxi Medical Journal* 2018; **40**.

20 Beauchamp LC, Nichols KR, Knoderer CA. Outcomes of Extended Infusion Cefepime in Pediatric Patients. *Infectious Diseases in Clinical Practice* 2019; **27**: 283–7.
